# Synthesis, Spectroscopy and Electrochemistry in Relation to DFT Computed Energies of Ferrocene- and Ruthenocene-Containing β-Diketonato Iridium(III) Heteroleptic Complexes. Structure of [(2-Pyridylphenyl)_2_Ir(RcCOCHCOCH_3_]

**DOI:** 10.3390/molecules24213923

**Published:** 2019-10-30

**Authors:** Blenerhassitt E. Buitendach, Jeanet Conradie, Frederick P. Malan, J. W. (Hans) Niemantsverdriet, Jannie C. Swarts

**Affiliations:** 1 Department of Chemistry, University of the Free State, Bloemfontein 9300, South Africa; blenerbuitendach@gmail.com (B.E.B.); conradj@ufs.ac.za (J.C.); 2 Department of Chemistry, University of Pretoria, Pretoria 0002, South Africa; frikkie.malan@gmail.com; 3 SynCat@DIFFER, Syngaschem BV, De Zaale 20, 5612 AJ Eindhoven, The Netherlands; J.W.Niemantsverdriet@tue.nl

**Keywords:** iridium, ferrocene, ruthenocene, betadiketone, substituent effects, crystal structure, electrochemistry, spectroelectrochemistry, phosphorescence, electronic spectrum

## Abstract

A series of new ferrocene- and ruthenocene-containing iridium(III) heteroleptic complexes of the type [(ppy)_2_Ir(RCOCHCOR′)], with ppy = 2-pyridylphenyl, R = Fc = Fe^II^(η^5^-C_5_H_4_)(η^5^-C_5_H_5_) and R′ = CH_3_ (**1**) or Fc (**2**), as well as R = Rc = Ru^II^(η^5^-C_5_H_4_)(η^5^-C_5_H_5_) and R′ = CH_3_ (**3**), Rc (**4**) or Fc (**5**) was synthesized *via* the reaction of appropriate metallocene-containing *β*-diketonato ligands with [(ppy)_2_(μ-Cl)Ir]_2_. The single crystal structure of **3** (monoclinic, P2_1_/n, Z = 4) is described. Complexes **1**–**5** absorb light strongly in the region 280−480 nm the metallocenyl β-diketonato substituents quench phosphorescence in **1**–**5**. Cyclic and square wave voltammetric studies in CH_2_Cl_2_/[N(^n^Bu)_4_][B(C_6_F_5_)_4_] allowed observation of a reversible Ir^III/IV^ redox couple as well as well-resolved ferrocenyl (Fc) and ruthenocenyl (Rc) one-electron transfer steps in **1**−**5**. The sequence of redox events is in the order Fc oxidation, then Ir^III^ oxidation and finally ruthenocene oxidation, all in one-electron transfer steps. Generation of Ir^IV^ quenched phosphorescence in **6**, [(ppy)_2_Ir(H_3_CCOCHCOCH_3_)]. This study made it possible to predict the Ir^III/IV^ formal reduction potential from Gordy scale group electronegativities, χ_R_ and/or Σχ_R′_ of β-diketonato pendent side groups as well as from DFT-calculated energies of the highest occupied molecular orbital of the species involved in the Ir^III/IV^ oxidation at a 98% accuracy level.

## 1. Introduction

Despite the scarcity and high price of iridium, an important commercial use of this metal and its complexes is the Cativa^TM^ process for acetic acid generation by carbonylation of methanol [1,2]. However, large scale commercial iridium-catalysed generation of hydrogen by water electrolysis is hindered by the scarceness and high cost of the metal. During electrolysis, iridium catalyses the oxygen evolution half reaction [3,4]. Although homoleptic tris(*β*-diketonato)iridium(III) complexes are almost unheard of in literature with only a handful of reports available [5,6], heteroleptic Ir(III) complexes containing a chelating *β*-diketonato ligand in addition to N- and C- bonding ligands have been extensively studied [7]. Complexes incorporating heavy transition metals such as platinum [8], osmium [9] and iridium [10,11] have been employed as emitters in electroluminescence devices, of which the iridium complexes are generally the most effective. The extensively studied organic light emitting iridium complex, *fac*-tris(2-pyridylphenyl-N,C^2′^)iridium(III), [Ir(ppy)_3_], has C^∧^N cyclometalate ligands [12]. This anionic ligand offers a strong Ir-C covalent interaction and, consequently, exhibits a highly stabilized ligand-field strength. Metallocene-containing *β*-diketonato complexes of Ir(III) are hitherto unknown.

Ferrocene-containing *β*-diketones, FcCOCH_2_COR, have been synthesized and characterized [13] before, as well as tested for cytotoxic activity [14] The ruthenocene analogues of these ligands have also been described [15] and tested for cytotoxicity [16]. While there are many homometallic *β*-diketonato complexes known as precursors for the preparation of oxide materials [17], heterometallic *β*-diketonato complexes are less common [18,19] although they are known since the seventies [20]. Especially 1-ferrocenyl-1,3-butanedione have been used to generate bi-, tri- and multi nuclear complexes of Re [21], Ni, Pd [22], Pr, Eu, Gd, Tb, Dy, Ho [23], Sm, Er and Yb [24]. These multi nuclear complexes often shows reversible electrochemistry with resolved ferrocenyl redox processes as shown for Hf and Zr phthalocyaninato complexes [25] as well as for octahedral tris(*β*-diketonato)aluminium(III) [26] and tris(*β*-diketonato)-manganese(III) [27] complexes. Cyclic voltammetric measurements showed these [M(FcCOCHCOR)_3_] complexes (M = Al or Mn) also display improved electronic communication between pendent *β*-diketonato substituents compared to [Cu(FcCOCHCOR)_2_] complexes [28]. Strong electronic communication between *β*-diketonato R substituents and the metal to which they are co-ordinated even influence kinetic parameters: square planar rhodium *β*-diketonato complexes showed their reactivity towards oxidative addition or substitution respectively is enhanced several orders of magnitude respectively by electron-donating *β*-diketonato R side groups [29,30]. Similarly, density functional theory (DFT) studies on the substitution of *β*-diketonato-1,5-cyclooctadieneiridium(I) complexes with 1,10-phenanthroline showed linear relationships between calculated orbital energies and charges of [Ir(*β*-diketonato)(cod)] complexes as well as the experimental second order substitution rate constant of the reaction [31].

We report here the synthesis, spectroscopy and electrochemical characterization of new ferrocene- and ruthenocene-containing iridium(III) heteroleptic complexes [(ppy)_2_Ir(RCOCHCOR′)], with R = Fc = Fe^II^(η^5^–C_5_H_4_)(η^5^–C_5_H_5_) and Rʹ = CH_3_ (**1**), Fc (**2**), as well as R = Rc = Ru^II^(η^5^–C_5_H_4_)(η^5^–C_5_H_5_) and R′ = CH_3_ (**3**), Rc (**4**) and Fc (**5**) and also R = R′ = CH_3_ (**6**); (ppy = 2-pyridylphenyl). Iridium(III) oxidation potentials are also related to DFT-calculated HOMO energy levels of the species involved. 

## 2. Results and Discussion

### 2.1. Synthesis 

**Scheme 1:**
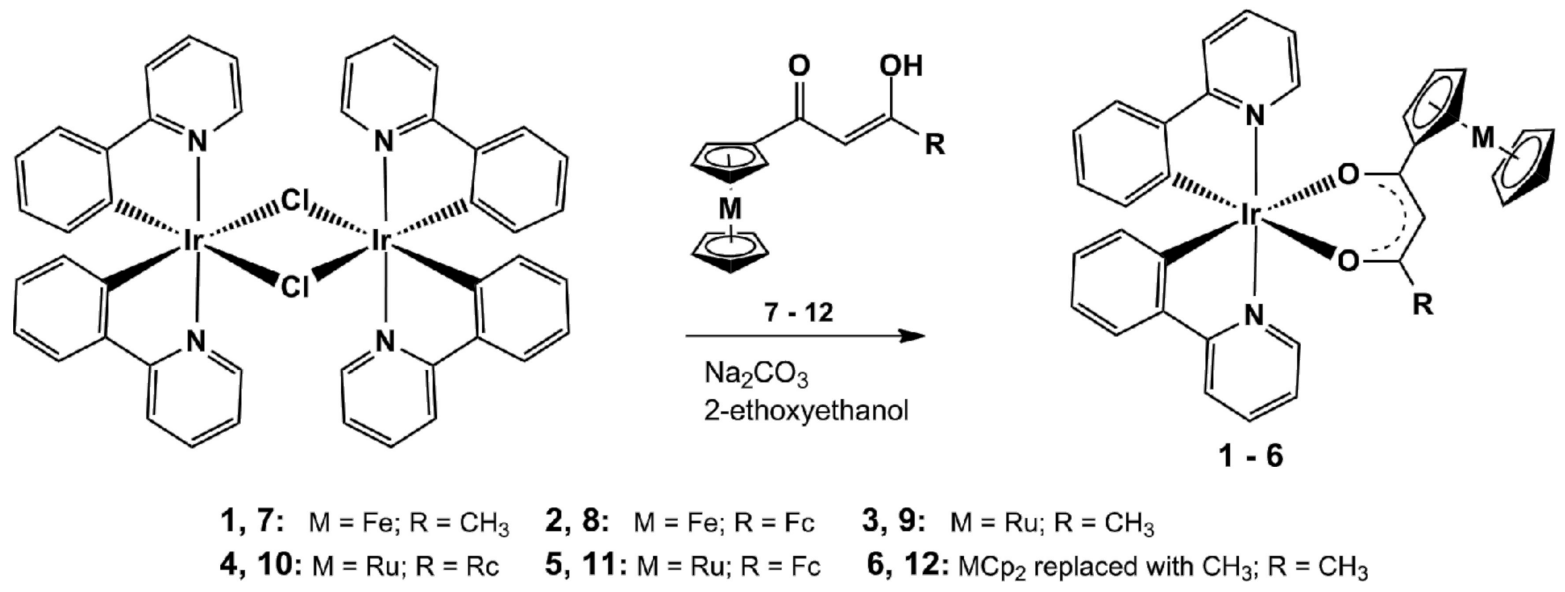
Synthesis of **1**–**6** from the chloro-bridged dimeric iridium(III) complex di-μ-chlorotetrakis[2-(2-pyridinyl-*k*N)phenyl-*k*C]diiridium(III). *Trans* isomers with respect to N-atoms of the ppy-ligands of **1**–**6** are shown as the single crystal X-ray structure determination, see below, confirms this geometric orientation. Fc = ferrocenyl, Rc = ruthenocenyl and Cp = cyclopentadienyl = C_5_H_5_^−^.

The metallocene-containing iridium(III) heteroleptic complexes [(ppy)_2_Ir(RCOCHCOR′)], **1**–**6**, were prepared as shown in Scheme 1 *via* adaption of published methods [32] to be applicable to synthesis of the present new complexes. The chloride-bridged dimer [(ppy)_2_(μ-Cl)Ir]_2_ was readily converted to the monomeric complexes by reacting it with the bidentate, monoanionic metallocene-containing *β*-diketonato ligands RCOCH_2_COR′ where R = Fc and R′ = CH_3_ (**7**), Fc (**8**), as well as R = Rc and R′ = CH_3_ (**9**), Rc (**10**) and Fc (**11**) and also R = R′ = CH_3_ (**12**). 

After work-up, which included column chromatography and recrystallization, the new air-stable heteroleptic iridium(III) complexes **1**–**5** could be isolated as solids, ranging from yellow (compound **1**) to orange-red (compounds **2**, **5**), in moderate (30–70%) yields. Complexes **1**–**5** are soluble in most organic solvents and crystallographic quality needle-like crystals were obtained from dichloromethane:*n*-heptane = 1:1. For comparison purposes the known acetylacetonato derivative [(ppy)Ir(CH_3_COCHCOCH_3_)] (**6**) was also synthesized.

### 2.2. Single Crystal X-Ray Structure of 3 

**Figure 1:**
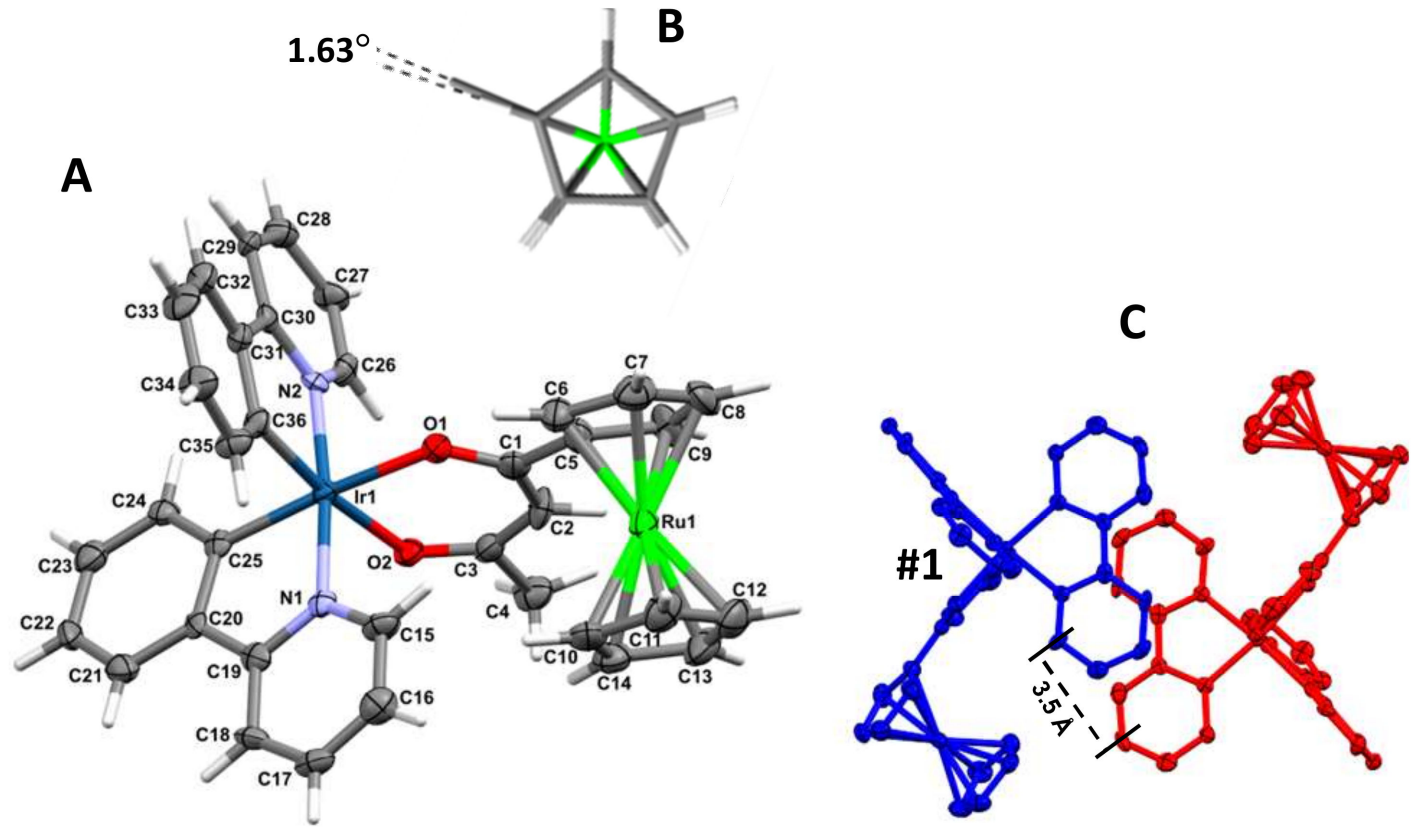
**Left, A:** Molecular structure of [(ppy)_2_Ir(RcCOCHCOCH_3_)] (**3**) showing atom labeling and, **B**, the ruthenocenyl group from above highlighting the near-eclipse configuration. The thermal ellipsoids represent a 50% probability limit. For clarity, a co-crystallized molecule of CH_2_Cl_2_ have been omitted. **Right: C** shows the crystal packing for **3**, with the foreground molecule labelled **#1** in blue and viewed perpendicular to the ppy ligand plane of the molecule (red) in the background. The perpendicular π–π spacing between the ppy ligands of adjacent molecules is ca. 3.5 Å. Selected bond distances (angstroms) are: Ir(1)–N(1) 2.046(7), Ir(1)–N(2) 2.045(7), Ir(1)–C(36) 1.987(8), Ir(1)–C(25) 1.992(9), Ir(1)–O(1) 2.112(6), Ir(1)–O(2) 2.158(6), O(1)–C(1) 1.262(10), O(2)–C(3) 1.250(11), C(1)–C(2) 1.402(14), C(2)–C(3) 1.393(14), C(1)–C(5) 1.500(14), C(3)–C(4) 1.528(13); Selected bond angles (degrees): N(1)–Ir(1)–C(25) 80.4(3), N(2)–Ir(1)–C(36) 80.8(3), O(1)–Ir(1)–O(2) 87.8(2), Ir(1)–O(1)–C(1) 126.1(6), Ir(1)–O(2)–C(3) 124.8(6), N(1)–Ir(1)–N(2) 174.3(3), O(1)–Ir(1)–C(25) 172.9(3), O(2)–Ir(1)–C(36) 172.3(3), O(1)–C(1)–C(2) 126.6(9), O(2)–C(3)–C(2) 127.4(8), C(1)–C(2)–C(3) 126.9(9), O(1)–C(1)–C(5) 113.9(9), O(2)–C(3)–C(4) 114.6(9). Other bond lengths and angles are available in the Supporting Information. Symmetry transformations used to generate equivalent atoms: #1 −x, −y, −z.

The structure of [(ppy)_2_Ir(RcCOCHCOCH_3_)] (**3**), was solved to determine the orientation of the ppy ligands relative to each other. Complex **3** crystallizes as yellow plates from CH_2_Cl_2_/n-heptane in the monoclinic space group P2_1_/n, with one molecule of **3** and a disordered CH_2_Cl_2_ (33% occupation) contained in the asymmetric unit cell. A molecular plot of **3** is shown in Figure 1 and crystallographic data are given in Table 1.

Complex **3** displayed a slightly distorted octahedral coordination geometry around the central Ir atom. Similar to [(ppy)_2_Ir(CH_3_COCHCOCH_3_)], **6**, [32], it retains the *cis*-C,C′ *trans*-N,N′ chelate disposition of the chloride-bridged precursor dimer, [(ppy)_2_(μ-Cl)Ir]_2_ [33]. 

The average Ir–C bonds of (Ir–C_avg_ = 1.990(9) Å) are shorter than the Ir–N bonds (Ir–N_avg_ = 2.046(7) Å). These Ir–C bond lengths are comparable to values reported for the precursor dimer [(ppy)_2_(μ-Cl)Ir]_2_ (Ir–C = 1.986(3) − 2.008(7) Å) [33] and complex **6** (Ir−C_avg_ = 2.003(8) Å) [32]. Similarly, the Ir–N bond lengths (Ir–N_avg_ = 2.046(7) Å) also fall within the typical range of values reported for iridium complexes with ppy ligands, including **6** (Ir–N_avg_ = 2.021(7) Å), but shorter than the average length of 2.140 (5) Å observed in *fac*-Ir(ppy)_3_ [34]. 

The Ir(1)–O(1) and Ir(1)–O(2) bond lengths of 2.112(6) and 2.158(6) Å respectively are notably longer than the mean Ir–O value of 2.088 Å reported for related iridium compounds in the Cambridge Crystallographic Database [35]. Since the Ir–O bond lengths are within 4σ(I) Å the same, coordination of the *β*-diketonato ligand to the Ir core is considered to approach a symmetric coordination sphere. All other bond lengths and angles within the chelate ligands are typical for cyclometalated ppy and acetylacetonato ligands bound to Ir(III).

Unconjugated C=O bond lengths in β-diketones are typically 1.206 Å, while C−O bond lengths are 1.300 Å [36,37]. For **3**, the C−O bond lengths are between these C=O and alkoxy bond length extremes with C(1)−O(1) = 1.262(10) Å and C(3)−O(2) = 1.250(11) Å. For **3** the difference between lengths of the C−O bonds is 0.012(15) Å, while the difference between unconjugated C=O and C−O bonds in β-diketones is 0.094(10) Å. The C−O bonds encountered in **3** are thus indicative of significant delocalized character in the *β*-diketonato fragment. Bond lengths C(1)–C(2) and C(2)–C(3) are within 1σ(I) the same (Figure 1) implying the *β*-diketone is symmetric.

Regarding the ruthenocenyl groups, the average C−C bond length within the ruthenocenyl group is 1.414(16) Å for the unsubstituted cyclopentadienyl (Cp) rings and 1.420(17) Å for substituted cyclopentadienyl rings. The largest deviations from this average are +0.016(23) Å for the C(12)−C(13) bond and −0.016(22) Å for the C(5)−C(9) bond. Bond angles in the unsubstituted and substituted cyclopentadienyl rings averaged 108°, which is the ideal theoretical value. The largest deviation from the average values was for the C(10)−C(14)−C(13) angle (+1.70910)^o^) on the unsubstituted Cp ring. The ruthenocenyl group thus exhibit the expected normal delocalized bond lengths and angles. Cp-rings of the ruthenocenyl group were found to exist almost exactly in the eclipsed configuration. The deviation from a complete eclipsed conformation, as measured with the dihedral angle C(14)−cent(Cp-ring)−cent(subst-Cp-ring)−C(5) was only ca. 1.63° (cent = centroid), Figure 1. Deviation of the two Cp-rings from parallel was measured at ca. 1.15° while deviation of the substituted Cp-ring from the *β*-diketonato chelate plane Ir-O(1)-C(1)-C(2)-C(3)-O(2) was measured at ca. 18.97^o^. 

The crystal packing of **3** shows similar ppy-ligand positioning to that of **6**, with a nearest neighbour molecule related by a C2 rotation that positions a ppy-ligand staggered relative to that of adjacent molecules, as shown in Figure 1 right. For both **3** and **6**, the distance between adjacent ring planes of the aromatic ring systems was measured as ca. 3.5 Å face-to-face separation, and a centroid(arene)-centroid(pyridyl) distance of 4.53 Å (at an angle of 5.65°), falls within the range for classification of weak π–π interactions [34]. 

### 2.3. Spectroscopic Studies 

The IR spectra of **1**–**6** showed the characteristic strong *ν*(C=O) vibrations found between 1510–1562 cm^−1^ (Materials and Methods section) which is typical for chelate-bonded β-diketonato ligands in transition metal complexes [38,39]. Generally, a shift to lower wave numbers is observed for **1**–**6** compared to the free *β*-diketones **7**–**12** [40,41], which allows monitoring of the reaction progress. 

**Figure 2:**
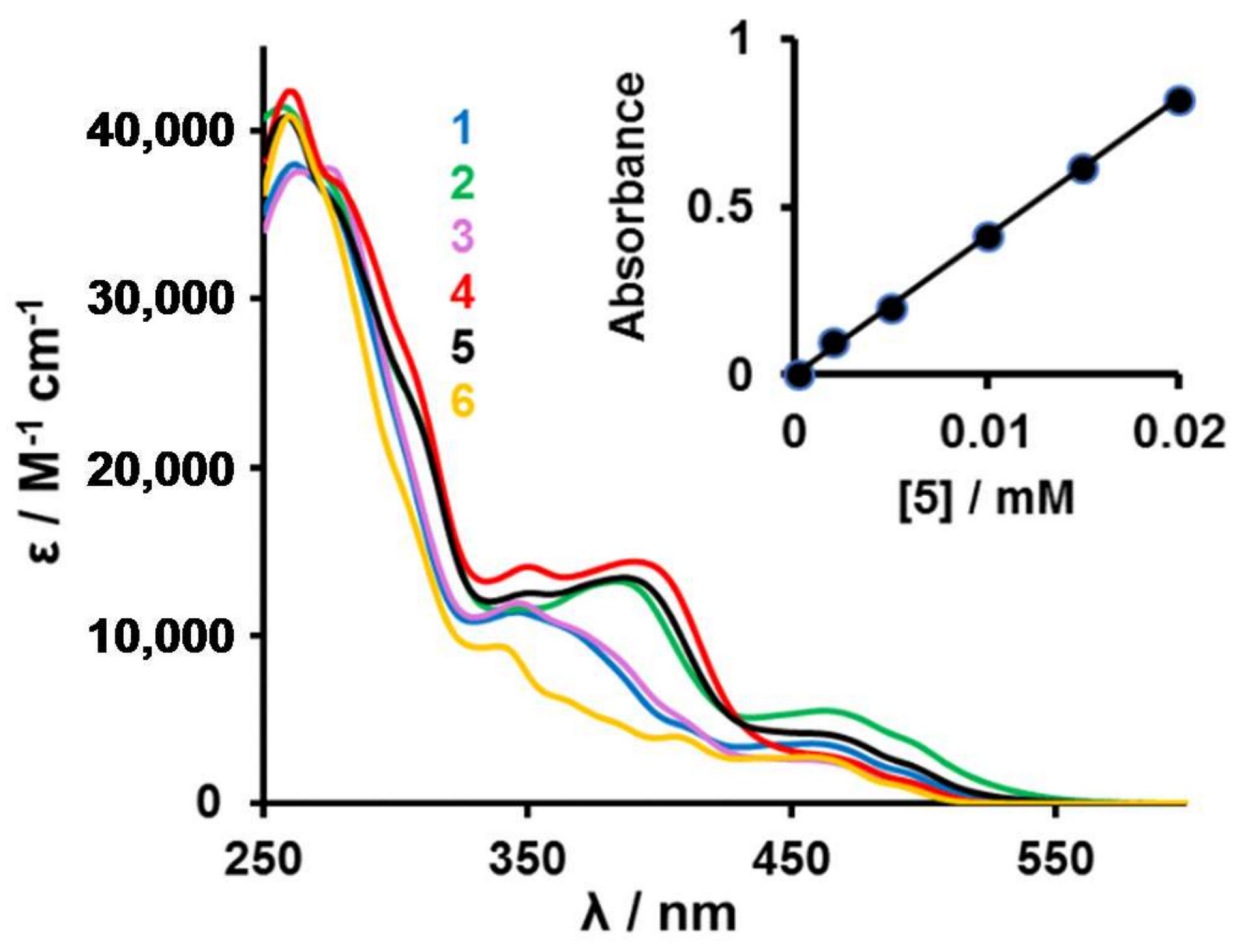
UV–Vis spectra of [(ppy)_2_Ir(RCOCHCOR′)] complexes **1** (R = Fc, R′ = CH_3_, blue), **2** (R = R′ = Fc, green), **3** (R = Rc, R′ = CH_3_, purple), **4** (R = R′ = Rc, red), **5** (R = Fc, R′ = Rc, black) and **6** (R = R′ = CH_3_, orange) in CH_2_Cl_2_. *Insert*: The linear relationship between absorbance and concentration for complex **5** at λ = 262 nm.

The UV–Vis absorption spectra of complexes **1**–**5** in CH_2_Cl_2_ solutions at room temperature are shown in Figure 2 and peak maxima are summarized in Table 2. Each absorption spectrum is composed of an intense absorption band in ultraviolet region and a weaker absorption band in visible region. In analogy to other studies, the higher energy absorption bands below 300 nm are assigned to spin-allowed intraligand π-π* transitions (corresponding to ligand centred states, ^1^LC), while the lower energy absorption bands between 340 and 540 nm are assigned to a mixture of metal to ligand charge transfers (^1^MLCT and ^3^MLCT) [10,11,12,42]. A linear relationship between absorbance and concentration (insert Figure 2) indicated all complexes followed the Beer-Lambert law, A = εC*l*. 

In the wavelength region 355 ≤ λ ≤ 430 nm, the influence of the number of metallocenyl substituents on absorption bands is observable. Each of the complexes **2**, **4** and **5** has two metallocenyl groups in the β-diketonato ligand coordinated to the iridium centre. These complexes exhibit relatively strong absorbance bands with λ_max_ at 384, 390 and 386 nm (ε > 13200 M^−1^·cm^−1^) grouped closely together. The same absorption bands in complexes **1** and **3**, which have only one metallocenyl substituent, became much weaker and was shifted to shorter wave lengths. It is only observable as a shoulder at ca. 371 nm (ε = 9010 M^−1^·cm^−1^). For complex **6**, which has no metallocene in its structure, the comparable absorption band was barely detectable as a weak shoulder at 363 nm (ε = 6330 M^−1^·cm^−1^).

### 2.4. Electrochemistry

**Figure 3:**
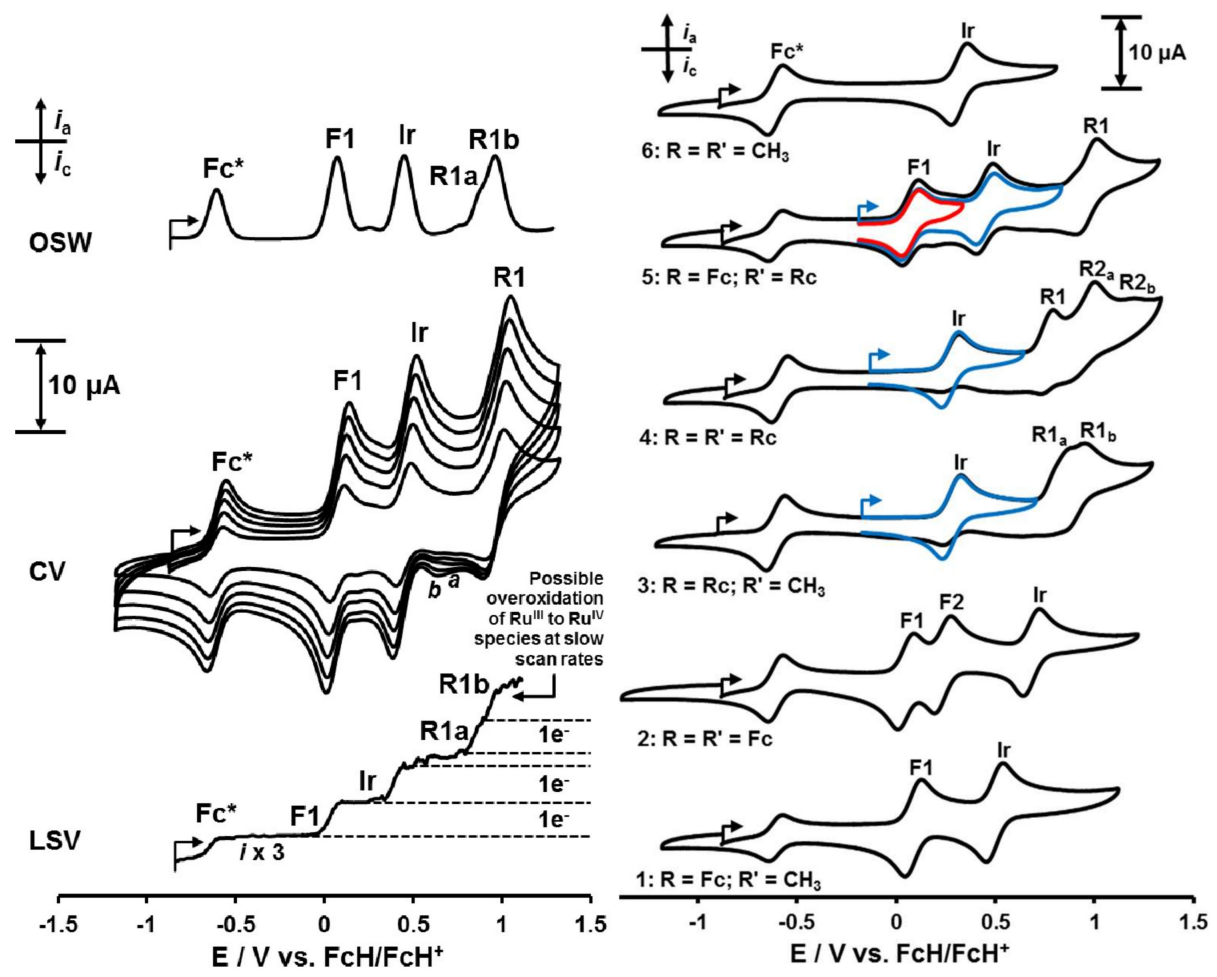
**Left**, Top: OSW (Osteryoung Square Wave) voltammogram of 0.5 mmol·dm^−3^ solutions of [(ppy)_2_Ir(FcCOCHCORc)], 5, in CH_2_Cl_2_ (20 ^o^C) at 10 Hz in the presence of Fc* = decamethylferrocene as internal standard. **Left**, Middle: Cyclic voltammograms at scan rates 0.1 (smallest currents), 0.2, 0.3, 0.4 and 0.5 V·s^−1^ (largest current). **Left**, Bottom: LSV (Linear Sweep Voltammogram) at 2 mV·s^−1^. The ferrocenyl-, iridium- and ruthenocenyl-based redox processes are labelled as F1, Ir and R1 respectively. The small CV reduction peaks labeled “a” and “b” may be associated with either the presence of a Ru^IV^ species, or more likely a dimeric Ru^III^ ruthenocenium species [43]. Likewise, the LSV peak R1b counting 1 electron is consistent with Ru^IV^ formation on LSV timescale. **Right**: Cyclic voltammograms of ca. 0.5 mmol·dm^−3^ solutions of [(ppy)_2_Ir(RCOCHCORʹ)] complexes **1**–**6**, scanned at 100 mV·s^−1^ in CH_2_Cl_2_ at 20 ^o^C in the presence of 0.1 mol·dm^−3^ [N(^n^Bu)_4_][B(C_6_F_5_)_4_] as supporting electrolyte. Decamethylferrocene, Fc*, was used as internal reference, it is identified by the wave at -0.608 V *vs.* FvH/FcH^+^. Blue and red inserts: CV scans of 3, 4 and 5 where scan directions are reversed before ruthenocene oxidation commenced.

Cyclic voltammetry (CV), linear sweep voltammetry (LSV), and Osteryoung square wave voltammetry (OSW) were conducted on 0.5 mM solutions of iridium(III) complexes **1**–**6** in dry CH_2_Cl_2_ utilizing 0.1 mol·dm^−3^ [(^n^Bu)_4_N][B(C_6_F_5_)_4_] as supporting electrolyte. Data for cyclic voltammetry experiments are summarized in Table 3, and CVs of **5** at different scan rates are shown in Figure 3 (left) while Figure 3 (right) enables comparison of CV’s of **1**−**6** at a scan rate of 100 mV·s^−1^ with each other. CH_2_Cl_2_ was used as solvent because it minimizes solvent−compound interactions, while the chosen supporting electrolyte, [(^n^Bu)_4_N][B(C_6_F_5_)_4_], minimizes ionic interactions of the type (cations)^n+^···^−^[B(C_6_F_5_)_4_] [15,43,44].

In this study, all studied iridium complexes **1**–**6** showed electrochemically reversible oxidation waves corresponding to the Fc^0/+^, Ir^III/IV^ and Rc^0/+^ redox couples, although the Rc^0/+^ couple was not chemically reversible. One-electron electrochemical reversible redox processes are theoretically characterized by CV peak separations of ΔE_p_ = E_pa_ − E_pc_ = 59 mV [45,46]. Chemical reversibility is exhibited by *i*_pc_/*i*_pa_ current ratios approaching unity. 

The first redox process observed in the electrochemical sequence of redox events of **1**–**6** is the ferrocenyl oxidation wave labeled F1 in the CV’s of **1**, **2** and **5** (Figure 3). ΔE for all these redox processes was 82 mV or less. As the internal standard decamethylferrocene, Fc*, showed ΔE = 77 mV (Table 3), waves F1 in all cases were regarded as electrochemical reversible as the Fc* redox process at slow (100 mV·s^−1^) scan rates. Current ratios for wave F1 of **1** and **2** approached unity (Table 3) but for **5** it was only 0.55 at 100 mV s^−1^ scan rate. This indicated that, unlike for **1** and **2**, wave F1 for **5** was not chemically reversible. However, if the reversing potential was set to exclude redox activity of the ruthenocenyl couple, (blue and red inserts Figure 3, right), the ferrocenyl wave current ratio also approached 1. 

It is concluded that the unstable and highly reactive ruthenocenium species, Rc^+^ that is generated when the ruthenocenyl group is oxidized [43], probably interacts with the ppy ligands of **5** in such a way that the molecule degrades, thereby interfering with the reversibility of the Fc/Fc^+^ couple. Formal reduction potentials, E^o^′ = ½(E_pa_ + E_pc_), could therefore be calculated [47,48] for wave F1 of **1**, **2** and **5** and are summarized in Table 3. F1 E^o^ʹ values for **1**, **2** and **5** deviate from 0 mV vs. FcH/FcH^+^ in the range 50 ≤ E^o^′_Fc of Ir complex_ ≤ 86 mV, Table 3. This is a smaller deviation than for the Mn(FcCOCHCOR)_3_ [27], Cu(FcCOCHCOR)_2_ [28] or the free *β*-diketones FcCOCH_2_COR [13] which exhibited 52 ≤ E^o^′_Fc group of Mn_ ≤ 97 mV, 98 ≤ E^o^′_Fc group of Cu_ ≤ 233 mV and 188 ≤ E^o^′_Fc group free *β*-diketone_ ≤ 236 mV, respectively. Al(FcCOCHCOR)_3_ complexes exhibited a ferrocenyl reduction potential range of 33 ≤ E^o^′_Fc group of Al_ ≤ 86 mV [26]. These comparisons indicate that the electron-withdrawing effect that the *β*-diketone C=O group has on F1 ferrocenyl oxidation, moving it to larger (more cathodic) potentials, are much more negated by coordination to Ir^III^ than coordination to Mn^III^ or Cu^II^, but it is about the same as in Al^III^ coordination. 

Complex **2** has two ferrocenyl groups and the oxidation of the second ferrocenyl group is associated with wave F2 (Figure 3). This process is also electrochemically and chemically reversible at E^o^′ = 0.235 V vs FcH/FcH^+^. Upon oxidation of the ferrocenyl groups in complexes **1**, **2** and **5**, the electron-donating ferrocenyl group with group electronegativity [49,50], χ_Fc_ = 1.86 converts to an electron-withdrawing ferrocenium group with χ_Fc+_ = 2.82 (Table 2). The quantity “group electronegativity” also takes into account charge effects. The ferrocenium group is therefore almost as electron-withdrawing as the CF_3_ group which exhibits χ_CF3_ = 3.01. This strong electron-withdrawing property of Fc^+^ is the reason why wave F2 of complex **2** exhibits E^o^′ at a potential 185 mV larger than wave F1. The first-oxidized ferricenium group withdraws electron density *via* the conjugated β-diketonato backbone from the second still-to-be oxidized ferrocenyl group in **2** making this ferrocenyl group much more difficult to oxidize than the first one. The strong electron-withdrawing property of the Fc^+^ group also has a profound effect on the potential at which the Ir^III/IV^ couple is observed.

The next general redox process observed in the electrochemical sequence of redox events of **1**–**6** is the iridium wave labeled Ir in Figure 3 and Table 3 in the potential range E^o′^ **=** 0.252 − 0.681 V versus FcH/FcH^+^. For complex **6,** similar to that of the internal reference decamethylferrocene (Fc*), the iridium-based redox process was chemically and electrochemically reversible, displaying ΔE_p_ = 0.081 V and i_pc_/i_pa_ ratios approaching 1 at slow (100 mV·s^−1^) scan rates. A value of E^o′^ = 0.318 V was then calculated for **6** which is notably different than the published E^o′^ value [32] of 0.870 V versus Ag/AgCl (in DCM) or by subtracting 0.450 V [51] at 0.42V vs. FcH/FcH^+^ for **6**.

The Ir wave of complexes **1**–**6** displayed comparable ΔE values in the range 82–88 mV at 100 mV·s^−1^ scan rate, implying all Ir^III/IV^ couples approached electrochemical reversibility at slow (100 mV·s^−1^) scan rates. Only complexes **1** and **2** exhibited Ir^III/IV^ *chemical* reversibility with *i*_pc_/*i*_pa_ approaching 1 though, Table 3. However, when one sets the switching potential on anodic scans of **3**, **4** and **5** in such a way that the ruthenocenyl moiety is not oxidized at wave R, see blue inserts in Figure 3, right, then the Ir^III/IV^ couple of complexes **3**, **4** and **5** also becomes chemically reversible with current ratios approaching 1. It is clear that the highly reactive oxidized Rc^+^ fragments interact with the oxidized molecules as a whole and decomposes it to the extent that *i_pc_* for wave F1 of **5** and the Ir wave of **3**, **4** and **5** are smaller than stoichiometric reductions require. In the process, as explained before [15,43], a Ru^IV^ species probably irreversibly forms. In addition, all ruthenocene-containing complexes showed *i*_pc_/*i*_pa_ ratios also much less than one for the ruthenocenyl-based redox process implying the *i*_pc_ of the ruthenocenyl group waves is also much less than expected. This contrasts the electrochemistry of neat ruthenocene which do show *i*_pc_/*i*_pa_ current ratios of one in CH_2_Cl_2_/[(^n^Bu)_4_N][B(C_6_F_5_)_4_] [15]. The high reactivity of Rc^+^ is highlighted with the fact that even [(^n^Bu)_4_N][PF_6_] destroys it, generating a Ru^IV^ species [15,43]. The smaller-than-expected i_pc_ currents of waves F1, Ir and R in **3**, **4** and **5** are consistent with the unstable and highly reactive 17 electron ruthenicenium cation, Rc^+^, which forms in the anodic sweeps of compounds **3**, **4** and **5** at wave R, interacts with the fully oxidized molecular species and damage it. This explains why, when wave R is not initiated in the CV scans of **3**, **4** and **5**, waves F1 and Ir become also chemically reversible (i.e. *i*_pc_/*i*_pa_ ratios approach unity). 

The one-electron iridium-based redox process for complexes **1**–**5** have E^o′^ between 0.252–0.681 V versus FcH/FcH^+^ while for **6**, E^o′^ = 0.318 V (Table 3, Figure 3). The Ir redox process of **1**, **2** and, to a lesser extent **5**, are observed at potentials substantially larger than that of **6**. ΔE^o^′ = E^o^′**_1_**_, **5** or **2**_ − E^o^ʹ**_6_** = 364 (for **1**), 416 (for **5**) and 600 mV (for **2**). The reason for this large shift to bigger potentials is the electron-withdrawing nature of the oxidized ferrocenium group(s) in the *β*-diketonato ligand. E^o′^(Ir^III/IV^) of complex **2** is shifted to larger potentials than E^o′^(Ir^III/IV^) of **1** as the Ir center of **2** is exposed to two oxidized ferrocenium moieties when it becomes redox active. The Ir^III/IV^ couple of **5** is shifted the least because the ruthenocenyl group electronegativity is smaller than that of the CH_3_ group implying the ruthenocenyl group is more electron-donating than the CH_3_ group (χ_Rc_ = 1.89; χ_CH3_ = 2.34) [13,15]. Group electronegativity considerations, which also take into account charge changes on molecular fragments, therefore explain why the E^o^′ value of complex **5** is shifted the least, that of complex **1** intermediately and that of complex **2** the most to more positive values when comparing Ir^III/IV^couples of **1**, **2** and **5** with that of **6.** It also explains why E^o^′(Ir^III/IV^) of complexes **3** (R = CH_3_, R′ = Rc, E^o^′ = 0.283 V) and **4** (R = R′ = Rc, E^o^′ = 0.252 V) are shifted in the negative (cathodic) direction to smaller E^o^′ values than that of **6** having the CH_3_COCHCOCH_3_^−^ ligand (E^o^′ = 0.318 V) in the coordination sphere. The Ir center of complex **4** bearing two strongly electron-donating ruthenocenyl groups are oxidized the easiest in the present compound series **1**–**6**. 

In contrast to the ferrocenyl group’s reversible redox behavior of complexes **1**, **2** and **5**, irreversible electrochemistry was found for the ruthenocenyl centres of complexes **3**–**5**. These Rc-groups displayed comparable peak anodic currents, *i*_pa_, at peak anodic potentials, E_pa_, in the potential range 0.771–1.179 V in their cyclic voltammograms (waves R1 and R2 in Figure 3 as well as Table 3), while *i*_pc_, which is associated with E_pc_, were of very weak intensity. The chemical irreversibility of Rc-based redox processes is evident from the i_pc_/i_pa_ ratios of **3**–**5** ranging from 0.04 for R1a of complex **3** to 0.76 for R1 of complex **5**. Complex **3** shows two ruthenocenyl oxidation waves labelled R1_a_ and R1_b_ (Figure 3). Half wave R1_a_ is expected, but R1_b_ not. Similarly, two ruthenocene oxidations are expected for complex **4**, half waves R1 and R2a, but half wave R2_b_ is not expected. Complex **5** shows no anomalies for the ruthenocenyl anodic process, only anodic half wave R1 is observed at scan rate 100 mV·s^–1^. However, at a slow scan rate (LSV at 2 mV·s^–1^ and OSW at 10 Hz, (Figure 3, left) a hint of an unexpected wave R1_b_ is detected. A literature survey has shown that it is generally accepted that Ru^IV^ species arise in CV experiments involving ruthenocene derivatives in CH_3_CN/N(^n^Bu)_4_PF_6_ solvent/supporting electrolyte systems [15,43,52]. We conclude that traces of a Ru^IV^ species such as [(C_5_H_4_R)Ru^IV^(C_5_H_5_)]^2+^ are likely formed at wave R1_b_ or R2_b_ during the oxidation of the ruthenocenyl group, especially after Rc^+^ interacted with the ppy ligands of **3**–**5**. 

**Scheme 2:**
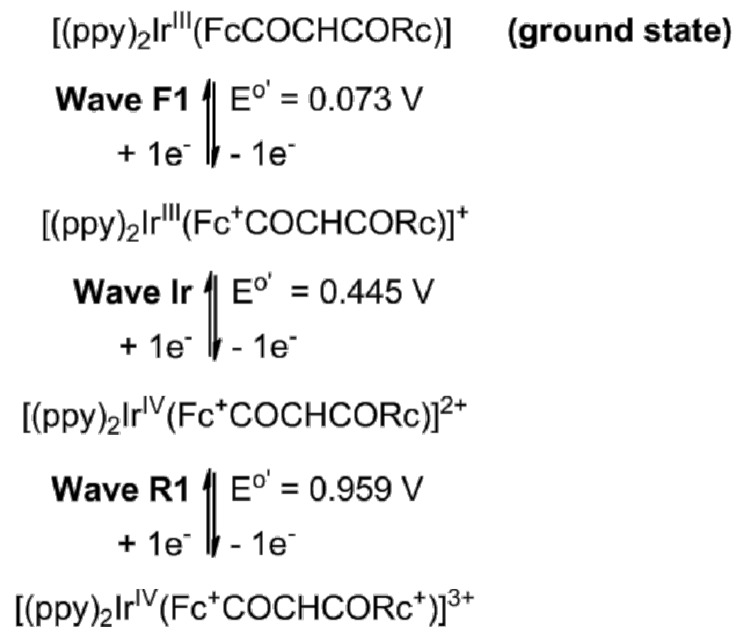
The sequence of electrochemical events associated with waves F1, Ir and R1 of [(ppy)_2_Ir(FcCOCHCORc)], 5. E^o^′ values are valid for a scan rate of 0.1 V·s^–1^.

The iridium, ferrocenyl and ruthenocenyl waves all involve the same number of electrons (one) transferred, as is demonstrated for **5** in Figure 3, left, with the LSV scan. With the above background, the sequence of electrochemical events is first ferrocenyl oxidation at wave F1, then iridium oxidation at wave Ir and then ruthenocenyl oxidation at wave R1. An electrochemical scheme describing the redox processes of **5** can be written, see Scheme 2.

Electrochemical schemes for **1**–**4** and **6** may be found in the Supplementary Information, and the sequence of redox events may also be deduced from the E^o^′ values in Table 3. 

### 2.5. Quantification of the Relationship between Σχ_R_ and E^o^′

**Figure 4:**
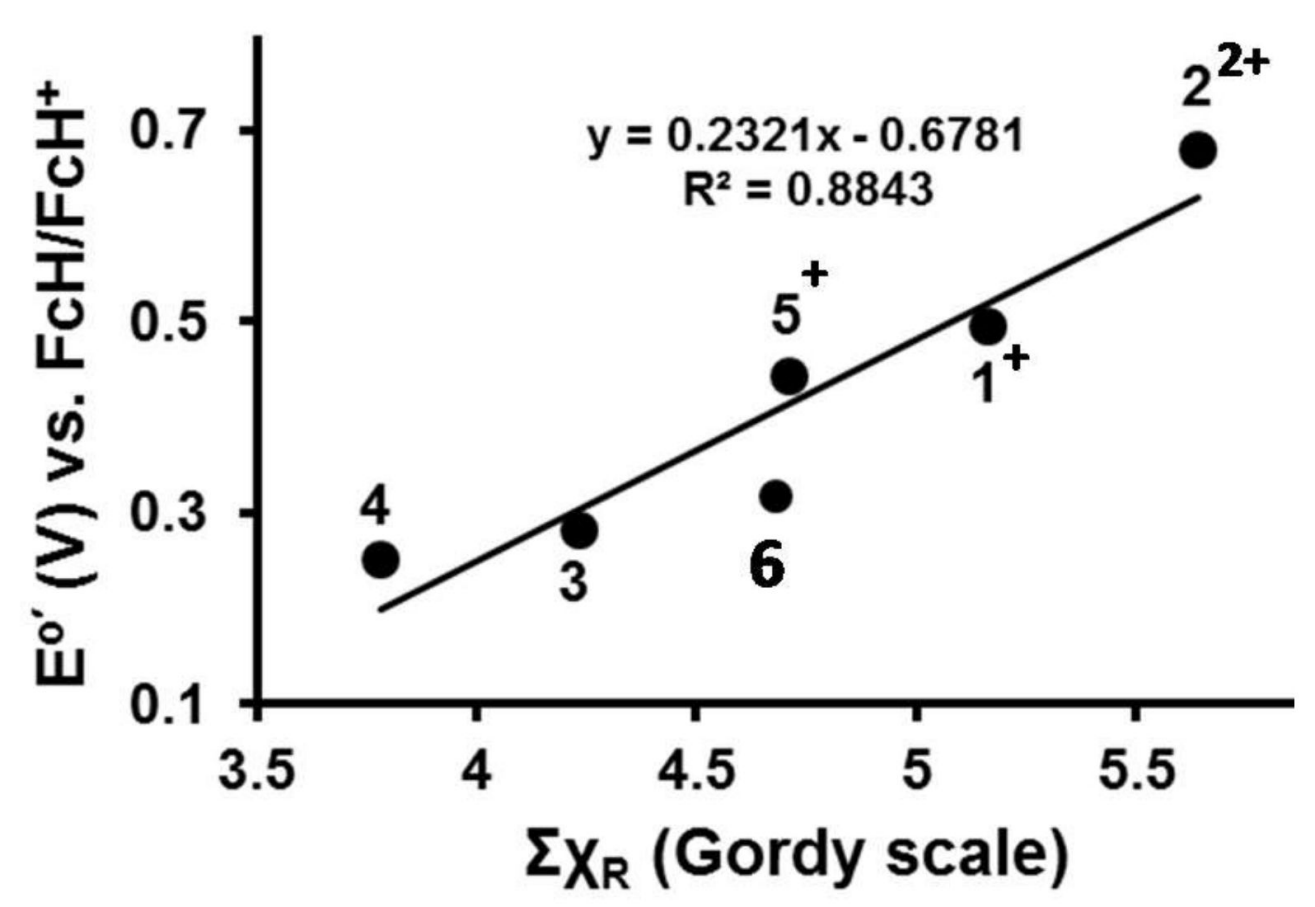
Linear relationship between the sum of the group electronegativities, Σχ_R_, and the iridium-based redox potentials, E°′, for compounds **1**–**6**.

The range of Ir^III/IV^redox potentials from 0.252 to 0.681 V vs FcH/FcH^+^ (Table 3) was traced to changes in χ_R_ values (χ_R_ values may be found in Table 2) of *β*-diketonato side groups Fc, Fc^+^, CH_3_, Rc and Rc^+^. As an example of how this is done, utilizing the sum of the R-group group electronegativities, Σχ_R_, of the *β*-diketonato ligand of **5** when Ir^III^ oxidation is initiated at wave Ir (Scheme 2 shows the reacting species for wave Ir as [(ppy)_2_Ir(Fc^+^COCHCORc)]^+^), Σχ_R_ may be calculated as Σχ_R_ = χ_Fc+_ + χ_Rc_ = 2.82 +1.89 = 4.71. In a similar way, Σχ_R_ may be calculated for all the *β*-diketonato ligands of **1**–**4** when Ir^III^ oxidation is initiated in the CV experiments. This data is plotted in Figure 4 showing the E^o^′ value of the Ir^III/IV^ couple is directly proportional to Σχ_R_ of the *β*-diketonato ligand.

A least square fitting of the data in Figure 4 allowed mathematical quantification as shown in Equation (1). The values in brackets are standard deviations.

E^o^′(Ir^III/IV^) = 0.23(4) Σχ_R_ – 0.68(20); R^2^ = 0.88 (1)

Equation (1) allows a rough estimation of the E^o^′ value of the Ir^III/IV^ couple in compounds of the type [(ppy)_2_Ir(RCOCHCOR′)] if the χ_R_ and χ_Rʹ_ values of *β*-diketonato R and R′ side groups are known.

### 2.6. DFT view of Iridium Oxidation

**Figure 5:**
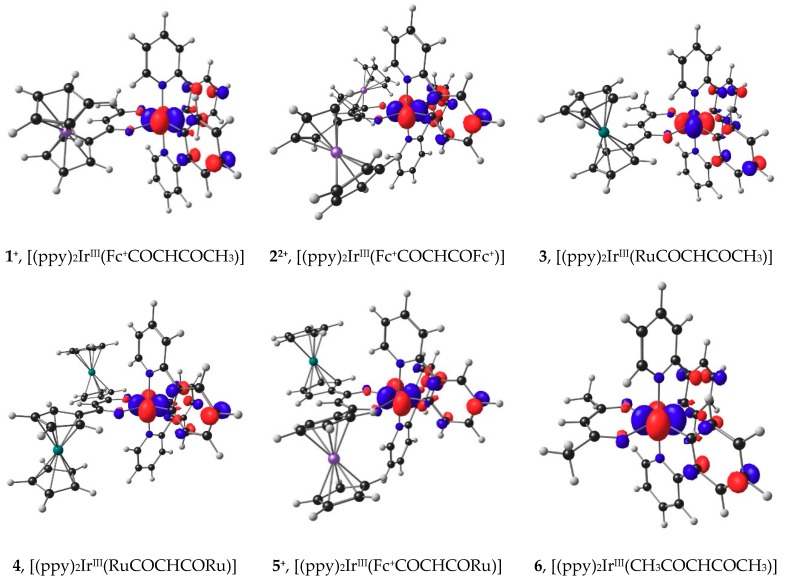
B3LYP/6-311G(d,p)/def2-TZVPP(SDD) calculated HOMOs of molecules involved in the Ir^III^ oxidation. Calculations was done using DCM as solvent. A contour of 0.06 eÅ^-3^ was used for the MO plots. Gas phase calculated HOMOs look similar and are provided in the Supporting Information Figure S2.

Oxidation of a molecule involves in most cases the removal of an electron from the highest occupied molecular orbital (HOMO) of the molecule [53]. The character of the HOMO thus gives the locus of the oxidation. The ease of oxidation is inversely proportional to the energy of the HOMO, since the larger the energy of the HOMO, the easier it will be to remove the electron at a lower redox potential. The HOMOs of molecules **3**, **4**, and **6** are iridium *d*_x2-y2_ based (see Figure 5) and the energy of the HOMOs, E_HOMO_, decreases as the E^o^′ values of ferrocenyl group oxidation (peak F1) increase (Data in Table S9 of the Supplementary Information).

For complexes **1**, **5** (both have only one ferrocenyl group in its structure) and **2** (this complex has two ferrocenyl groups in its structure) ferrocenyl oxidation occurs before the iridium center is oxidized. Thus, the HOMOs of the cations **1^+^** and **5^+^** will be associated with Ir^III^ oxidation, while the HOMO of the di-cation **2^2+^** is associated with iridium, see Figure 5. 

**Figure 6:**
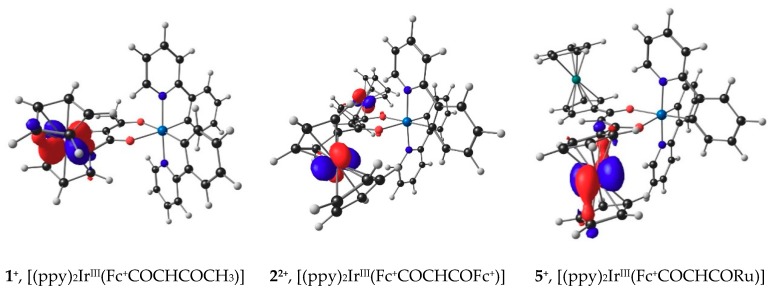
B3LYP/6-311G(d,p)/def2-TZVPP(SDD) calculated LUMOs of molecules after ferrocenyl oxidation. Calculations was done using DCM as solvent. A contour of 0.06 eÅ^–3^ was used for the MO plots. Gas phase calculated LUMOs look similar and are provided in the Supporting Information Figure S3.

**Figure 7:**
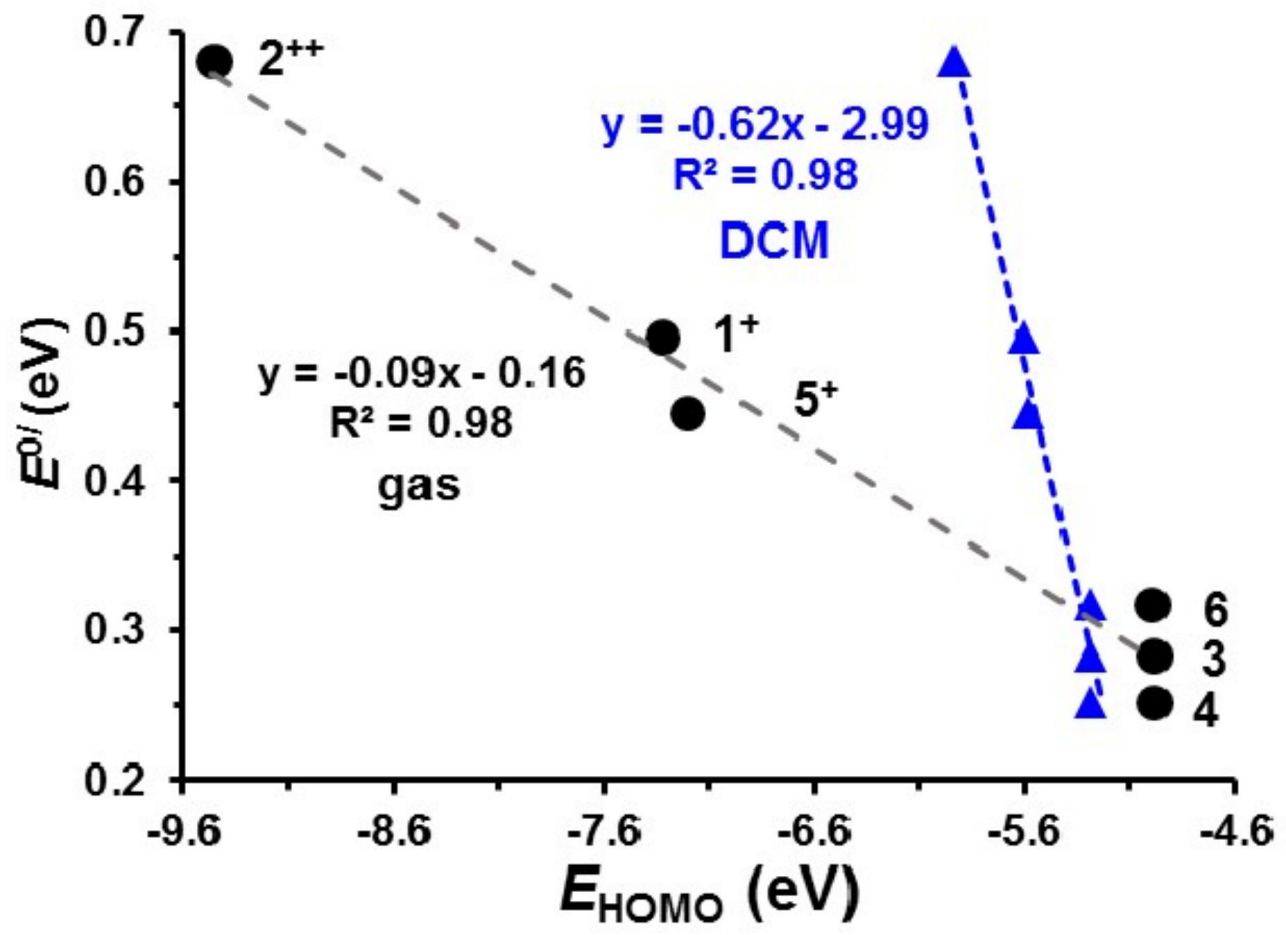
Relationship between the redox potential of the Ir^III/IV^ couple and the HOMO energy (*E*_HOMO_) of the species involved. The black data points were obtained by DFT calculations in gas phase, while the blue data points were obtained using DCM as solvent in the DFT calculations. Data in Table S9 of the Supporting Information.

The lowest unoccupied molecular orbitals (LUMO’s) of **1^+^**, **5^+^** and **2^2+^**, the orbitals where the electron(s) were removed during the initial ferrocenyl group oxidation, are located on iron, since ferrocenyl oxidation of **1**, **5** and **2** occur before iridium oxidation, as illustrated in Figure 6. The E^o^′ values of the Ir^III/IV^ couple of **1**–**6** relate to gas phase HOMO energies of **1^+^**, **2^2+^**, **3**, **4**, **5^+^** and **6** by Equation (2). Figure 7, black data points, shows an E^o^′ – E_HOMO_ plot of available gas phase data points. When taking the experimental solvent used for electrochemical analysis, namely dichloromethane, into account in the DFT calculations, a relationship to the same level of accuracy is obtained see equation 3, though the slope of the E^o^′ – E_HOMO_ plot is much steeper, as previously found when relating redox potentials with frontier orbital energies [54] (Figure 7, blue data points). 

Gas Phase: E^o^′ (Ir^III/IV^) = −0.087(7) E_HOMO_ − 0.16(5); R^2^ = 0.98 (2)

In DCM: E^o^ʹ (Ir^III/IV^) = −0.65(5) E_HOMO_ − 3.0(3); R^2^ = 0.98 (3)

In addition to Equation (1), equations (2) or (3) thus provide methods to estimate the E^o^′ value of the Ir^III/IV^ couple in compounds of the type [(ppy)_2_Ir(RCOCHCOR′)] by optimizing the most stable structure of the species involved in the iridium oxidation, using DFT calculations. E_HOMO_ of the optimized DFT calculated structure may then be obtained from the output DFT file.

### 2.7. Phosphorescence and Spectroelectrochemistry

**Figure 8:**
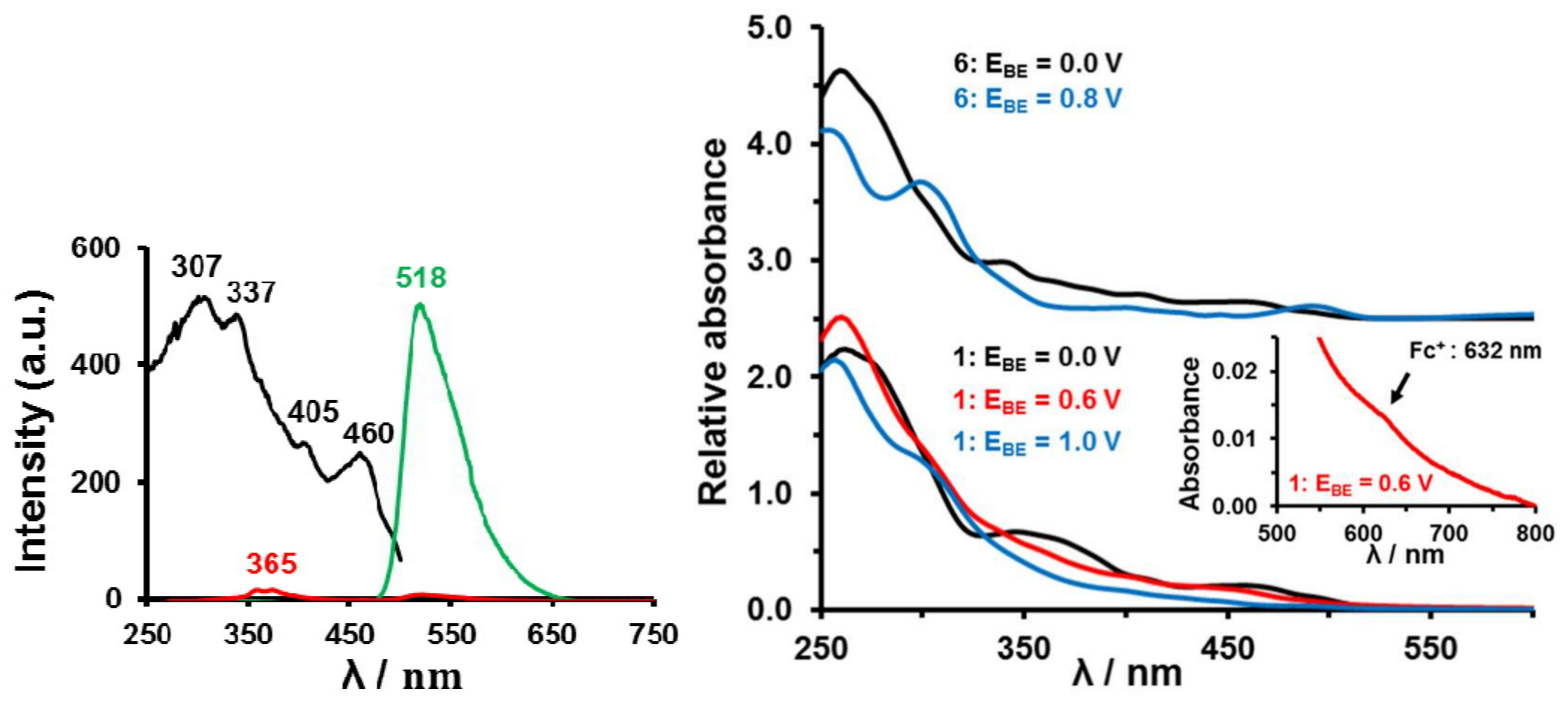
**Left:** Excitation spectrum (black) for observing emission at 518 nm and photoluminescence spectra (green and red) at 20 ^o^C of a CH_2_Cl_2_ solution containing 0.050 mmol dm^-3^ [(ppy)_2_Ir(acac)], **6**, and 0.05 mol dm^-3^ [N(^n^Bu)_4_][PF_6_], before (green) and after (red) bulk electrolysis at 0.8 V vs. Ag wire. After bulk electrolysis, Ir^III^ was converted to Ir^IV^, but the same excitation caused hardly any emission (red spectrum). **Right:** UV–Vis spectra of 0.05 mmol·dm^−3^ CH_2_Cl_2_ solutions of [(ppy)_2_Ir(acac)], **6**, (top) and [(ppy)_2_Ir(FcCOCHCOCH_3_)], **1**, (bottom) in CH_2_Cl_2_ in the presence of 0.05 mol·dm^−3^ [N(^n^Bu)_4_][PF_6_] at 20 ^o^C after bulk electrolysis (BE) at the indicated potentials (E_BE_ vs. Ag/Ag^+^). Black spectra are for **1** and **6** in the ground state. The red spectrum was obtained after Fc oxidation in **1** to generate **1^+^**; blue spectra are the result of Ir^III^ oxidation generating **1^2+^** or **6^+^**. The red insert highlights an electronic band associated with the Fc^+^ moiety of **1^+^**.

Complex **6** showed similar solid-state phosphorescence (irradiation with a UV-lamp at wavelengths 365 and 254 nm with a sample on a TLC plate) and CH_2_Cl_2_ solution photoluminescence as previously reported in literature [55] with an ambient temperature emission λ_max_ = 518 nm and lifetime of 1.1 μs, Figure 8. For **6** the lowest excited state is a triplet and as a result emission to the singlet ground state is forbidden without spin-orbit coupling (SOC). The emitting state is assigned as a ^3^MLCT (metal-to-ligand charge transfer) state and involves mainly occupied Ir 5d and unoccupied ppy π* orbitals [55]. The majority of complexes of the type [(C^N)_2_Ir(LX)] (where C^N = cyclometallating ligand such as ppy, and LX = monoanionic ligand such as acac) reported in literature showed efficient phosphorescence [10,11,12]. Upon performing bulk electrolysis (0.8 V vs. Ag wire) the Ir^III^ core is oxidized to Ir^IV^. Complex **6^+^** lost all phosphorescence, see Figure 8, left. This shows an Ir^III^ core is required for photoluminescence; Ir^IV^ quenches it. Figure 8, right, shows how the UV-Vis spectrum of **6** changed after bulk electrolysis converted it to **6^+^** having an Ir^IV^ core. The UV-vis spectra of **1**, **1^+^** and **1^2+^** are also shown.

The photophysical properties of complexes **1**–**5** are also quenched. Unlike the pale yellow [(ppy)_2_Ir(acac)] complex **6**, solid complexes **1**–**5** displayed hardly any phosphorescence when irradiated with a UV-lamp at wavelengths 365 and 254 nm. Neither did they display any phosphorescence in solution or after bulk electrolysis at 0.6 V vs. Ag wire (to oxidise Fc to Fc^+^ generating for example **1^+^**) and at 1.0 V (to oxidize Ir^III^ to Ir^IV^ generating **1^2+^**). Complexes **2**–**5** behaved in the same way showing that photoluminescence is quenched in **1**–**5** by the ferrocenyl, ferrocenium, ruthenocenyl and Ir^IV^ molecular fragments. Our results therefor mirror those of other researchers that indicated ruthenocenyl and ferrocenyl groups are photoluminescence quenchers [56].

## 3. Materials and Methods

### 3.1. General Information

Solid reagents ferrocene, ruthenocene (Sigma-Aldrich, Johannesburg, South Africa) and di-μ-chlorotetrakis[2-(2-pyridinyl-κN)phenyl-κC]diiridium(III) (STREM, Newburyport, MA, United States of America) were used without any further purification. Liquid reagents (Sigma-Aldrich and Merck, Johannesburg, South Africa) were used without any further purification unless specified otherwise. Dichloromethane was dried by distillation from calcium hydride and, to remove photochemically generated HCl, passed through basic alumina prior to use. For electrochemical experiments, spectrochemical grade dichloromethane (Aldrich) was used and stored under an argon atmosphere. Distilled water was used throughout. Metallocene-containing β-diketones FcCOCH_2_COR (**7**, R = CH_3_; **8**, R = Fc) [13] and RcCOCH_2_COR (**9**, R = CH_3_; **10**, R = Rc; **11**, R = Fc) [15] were synthesized utilizing published procedures with care being taken to separate it from FcCOCH=C(CH_3_)Fc, the aldol self-condensation product of acetyl ferrocene [57]. The complexes [(ppy)Ir(CH_3_COCHCOCH_3_)] (**6**, [32]) as well as tetrabutylammonium tetrakispentafluorophenyl-borate [58], and [N(^n^Bu)_4_][B(C_6_F_5_)_4_] were prepared as described before. Column chromatography was performed on Kieselgel 60 (Merck, grain size 0.040−0.063 nm) using hexane/diethyl ether (1:1) as mobile phase, unless otherwise specified. 

Infrared spectra were recorded on a Tensor 27 spectrophotometer (Bruker, Johannesburg, South Africa) equipped with a Bruker Platinum ATR accessory (diamond crystal), running OPUS software (Version 6.5) (Bruker, Johannesburg, South Africa). UV–Vis spectra were recorded on a Cary 5000 Probe UV-Vis-NIR spectrophotometer (Varian, Johannesburg, South Africa). Luminescence measurements were carried out on a Varian Cary Eclipse Fluorescence spectrometer. ^1^H-NMR spectra were recorded on a 300 MHz FOURIER NMR spectrometer or a 600 MHz AVANCE II NMR spectrometer (Bruker, Johannesburg, South Africa) operating at 25 °C with chemical shifts presented as δ values referenced to SiMe_4_ at 0.00 ppm utilizing CDCl_3_ as solvent. Melting points were determined using analytically pure samples on a BX51 microscope (Olympus, Johannesburg, South Africa) using a TMS 600 hot stage (LINKAM Johannesburg, South Africa). Elemental analyses were performed by Canadian Microanalytical Service (Delta, BC, Canada).

### 3.2. Synthesis 

The [(ppy)_2_Ir(RCOCHCOR′)] complexes **1**–**5** were prepared *via* modification of a published method [32] for synthesis of **6** to be compatible with complexes having metallocene-containing ligands. General procedure: A mixture of [(ppy)_2_(μ-Cl)Ir]_2_ (0.150 g, 0.14 mmol), the relevant *β*-diketone (**6**–**10**) (2.2 eq., 0.31 mmol) and sodium carbonate (2.2 eq., 0.31 mmol) were refluxed in 2-ethoxyethanol (7 mL) under inert atmosphere for 16 hours. The reaction mixture was allowed to cool to room temperature and the coloured precipitate was filtered off and washed with water and 10 mL of dry ethanol. The precipitate was then purified *via* chromatography on silica with a CH_2_Cl_2_/n-hexane/acetone (60:35:5) mobile phase. After removal of solvents, the product was dried under reduced pressure for 16 hours. Small crystallographic quality needle-like crystals were obtained for these complexes from slow evaporation of a CH_2_Cl_2_/n-heptane solution. 

### 3.3. Characterization Data

*bis(2-Phenylpyridinato-C2,N)(1-ferrocenylbutane-1,3-dione-κ^2^-O,O′)iridium(III)* (**1**)

R_f_: 0.83. Yield: 105 mg (0.14 mmol, 48% based on [(ppy)_2_(μ-Cl)Ir]_2_. ^1^H-NMR (300 MHz, CDCl_3_): *δ*, ppm 8.69 (d, 1H, *J* 5.8 Hz, ArH), 8.56 (d, 1H, *J* 5.8 Hz, ArH), 7.88 (d, 2H, *J* 7.7 Hz, ArH), 7.72 (m, 2H, ArH), 7.60 (d, 2H, *J* 7.8 Hz, ArH), 7.19 (m, 1H, ArH), 7.09 (m, 1H, ArH), 6.85 (m, 2H), 6.73 (m, 2H, ArH), 6.40 (dd, 1H, *J* 7.6, 1.2 Hz, ArH), 6.31 (dd, 1H, *J* 7.6, 1.1 Hz, ArH), 5.52 (s, 1H), 4.62 (m, 1H), 4.53 (m, 1H), 4.21 (m, 2H), 3.77 (s, 5H, FeC_5_H_5_), 1.89 (s, 3H). Calculated for C_36_H_29_FeIrN_2_O_2_: C, 56.18; H, 3.80; N, 3.64%. Found: C, 55.82; H, 3.78; N, 3.32%. M.p. >200 ^o^C (dec). IR: *ν*(CO) 1560 (s), 1546 (s), 1510 (vs) cm^−1^.

*bis(2-Phenylpyridinato-C2,N)(1,3-diferrocenylpropane-1,3-dionato-κ^2^-O,O′)-iridium(III)* (**2**)

R_f_: 0.76. Yield: 90 mg (0.10 mmol, 34% based on [(ppy)_2_(μ-Cl)Ir]_2_. ^1^H-NMR (300 MHz, CDCl_3_): *δ*, ppm 8.56 (dd, 2H, *J* 5.8, 0.9 Hz, ArH), 7.90 (d, 2H, *J* 8.0 Hz, ArH), 7.68 (m, 4H, ArH), 7.12 (m, 2H, ArH), 6.90 (td, 2H, *J* 7.4, 1.3 Hz, ArH), 6.77 (td, 2H, *J* 7.5, 1.3 Hz, ArH), 6.44 (dd, 2H, *J* 7.6, 1.0 Hz, ArH), 5.86 (s, 1H), 4.71 (m, 2H), 4.61 (m, 2H), 4.24 (t, 4H, *J* 1.9 Hz), 3.78 (s, 10H, 2 × FeC_5_H_5_). Calculated for C_45_H_37_Fe_2_IrN_2_O_2_: C, 57.39; H, 3.96; N, 2.97%. Found: C, 57.08; H, 3.71; N, 3.14%. M.p. > 250 ^o^C (dec). IR: *ν*(CO) 1542 (vs), 1510 (s) cm^−1^.

*bis(2-Phenylpyridinato-C2,N)(1-ruthenocenylbutane-1,3-dione-κ^2^-O,O′)iridium(III)* (**3**)

R_f_: 0.78. Yield: 126 mg (0.16 mmol, 55% based on [(ppy)_2_(μ-Cl)Ir]_2_. ^1^H-NMR (300 MHz, CDCl_3_): *δ*, ppm 8.60 (dd, 1H, *J* 5.9, 0.8 Hz, ArH), 8.50 (dd, 1H, *J* 5.9, 0.9 Hz, ArH), 7.88 (d, 2H, *J* 8.1 Hz, ArH), 7.74 (td, 2H, *J* 7.2, 1.6 Hz, ArH), 7.57 (dd, 2H, *J* 7.6, 3.3 Hz, ArH), 7.16 (m, 2H), 6.83 (m, 2H, ArH), 6.71 (m, 2H, ArH), 6.34 (dd, 1H, *J* 7.5, 1.1 Hz, ArH), 6.27 (dd, 1H, *J* 7.6, 1.0 Hz, ArH), 5.44 (s, 1H), 4.95 (m, 2H), 4.54 (t, 2H, *J* 1.8 Hz), 4.28 (s, 5H, RuC_5_H_5_), 1.84 (s, 3H). Calculated for C_36_H_29_IrN_2_O_2_Ru: C, 53.06; H, 3.59; N, 3.44%. Found: C, 53.16; H, 3.75; N, 3.14%. M.p. >200 ^o^C (dec). IR: *ν*(CO) 1561 (s), 1510 (vs) cm^−1^.

*bis(2-Phenylpyridinato-C2,N)(1,3-diruthenocenylpropane-1,3-dionato-κ^2^-O,O′)iridium(III)* (**4**)

R_f_: 0.89. Yield: 210 mg (0.20 mmol, 71% based on [(ppy)_2_(μ-Cl)Ir]_2_. ^1^H-NMR (300 MHz, CDCl_3_): *δ*, ppm 8.56 (dd, 2H, *J* 5.7, 0.8 Hz, ArH), 7.88 (d, 2H, *J* 7.9 Hz, ArH), 7.73 (td, 2H, *J* 7.6, 1.6 Hz, ArH), 7.59 (dd, 2H, *J* 7.7, 1.3 Hz, ArH), 7.15 (td, 2H, *J* 6.6, 1.4 Hz, ArH), 6.85 (td, 2H, *J* 7.4, 1.2 Hz, ArH), 6.72 (td, 2H, *J* 7.5, 1.3 Hz, ArH), 6.33 (dd, 2H, *J* 7.6, 0.9 Hz, ArH), 5.71 (s, 1H), 4.99 (m, 4H), 4.56 (m, 4H), 4.28 (s, 10H, 2 × RuC_5_H_5_). Calculated for C_45_H_37_IrN_2_O_2_Ru_2_: C, 52.36; H, 3.61; N, 2.71%. Found: C, 52.71; H, 3.27; N, 2.68%. M.p. > 250 ^o^C (dec.). IR: *ν*(CO) 1546 (vs), 1514 (s) cm^−1^.

*bis(2-Phenylpyridinato-C2,N)(1-ferrocenyl-3-ruthenocenyl-1,3-propanedionato-κ^2^-O,O′)iridium(III)* (**5**)

R_f_: 0.90. Yield: 84 mg (0.09 mmol, 32% based on [(ppy)_2_(μ-Cl)Ir]_2_. ^1^H-NMR (300 MHz, CDCl_3_): *δ*, ppm 8.63 (m, 2H, ArH), 7.89 (d, 2H, *J* 8.3 Hz, ArH), 7.71 (m, 2H, ArH), 7.62 (m, 2H, ArH), 7.14 (m, 2H, ArH), 6.87 (m, 2H, ArH), 6.74 (m, 2H, ArH), 6.38 (td, 2H, *J* 7.2, 0.9 Hz, ArH), 5.79 (s, 1H), 5.04 (t, 2H, *J* 1.7 Hz), 4.66 (m, 1H), 4.57 (m, 3H), 4.29 (s, 5H, RuC_5_H_5_), 4.22 (t, 2H, *J* 1.9 Hz), 3.77 (s, 5H, FeC_5_H_5_). Calculated for C_45_H_37_FeIrN_2_O_2_Ru: C, 54.76; H, 3.78; N, 2.84%. Found: C, 54.69; H, 3.42; N, 2.69%. M.p. > 250 ^o^C (dec.). IR: *ν*(CO) 1545 (vs), 1514 (s) cm^−1^.

### 3.4. Crystal Structure Determination of **3**

Single crystal diffraction studies on complex **3** were done using Quazar multi-layer optics monochromated Mo Kα radiation (k = 0.71073 Å) on a Bruker D8 Venture kappa geometry diffractometer (Bruker, Johannesburg, South Africa) with duo Iμs sources, a Photon 100 CMOS detector and APEX II control software [59]. X-ray diffraction measurements were performed at 150(2) K. Data reduction was performed using SAINT+ [59] and the intensities were corrected for absorption using SADABS [59]. The structures were solved by direct methods using *SHELXT* [60], using the *SHELXL-2014/7* [61] program. The non-hydrogen atoms were refined anisotropically. All H atoms were placed in geometrically idealised positions and constrained to ride on their parent atoms. For tables containing the data collection and refinement parameters, bond lengths and bond angles, see Tables S1–S8 in the Supplementary Information as well as CCDC 1916701.

### 3.5. Electrochemistry

Cyclic voltammograms (CV’s), square wave voltammograms (SW’s) and linear sweep voltamograms (LSV’s) were recorded using a computer-controlled BAS model 100 B potentiostat (Analytical Science Technology, Cape Town, South Africa). All experiments were performed in a dry cell under an argon atmosphere. Platinum wires were used for the pseudo internal reference electrode as well as the auxiliary electrode, while a glassy carbon working electrode (surface diameter 3.00 mm) was utilized. Between each set of scans the working electrode was polished on a Buhler polishing mat with 1 micron and then with ¼ micron diamond paste. All electrode potentials are reported using the potential of the ferrocene/ferrocenium redox couple FcH^+^/FcH (FcH = Fe^II^(η^5^–C_5_H_5_)_2_, E^o′^ = 0.00 V) as reference. Decamethylferrocene, Fc* [62], was used as internal standard to prevent signal overlap with ferrocenyls or iridium of complexes **1**–**5** (E^o′^ = −0.608 V versus free ferrocene with ΔE = 0.077 V and i_pc_/i_pa_ = 1 under the conditions employed). Analyte concentrations were ca. 0.5 mM in CH_2_Cl_2_ and 0.1 M tetrabutylammonium tetrakispenta- fluorophenylborate, [N(^n^Bu)_4_][B(C_6_F_5_)_4_] was used as supporting electrolyte. Data was exported to a spread sheet program for adjustment and diagram preparation. 

Bulk electrolysis (BE) experiments were performed using a computer-controlled BAS model 100 B potentiostat ((Analytical Science Technology, Cape Town, South Africa). BE experiments were conducted in a three-compartment cell with a Pt-mesh working electrode, Pt-wire auxiliary electrode and Ag-wire reference electrode. Solutions containing 0.05 mmol·dm^−3^ analyte and 0.05 mmol·dm^−3^ [N(^n^Bu)_4_][PF_6_] as electrolyte were subjected to a set potential until the current ratio reached 1.0 % of its original value.

### 3.6. DFT Calculations

All calculations were carried out using DFT in the gas or solvent phase using the Gaussian 16 program [63]. The B3LYP (Becke 1993 and Lee-Yang-Parr) hybrid functional [64,65] (UB3LYP for open shell species) with the triple-ζ basis set 6-311G(d,p) basis set was used for the lighter atoms (C, H, N, O, Fe), whereas the Stuttgart/Dresden (SDD) pseudopotential was used to describe the Ru and Ir electronic cores, while the metal valence electrons were described using the def2-TZVPP basis set [66]. For solvent calculations, the polarizable continuum model (PCM) [67] as implemented in Gaussian 16 was used with dichloromethane as solvent.

## 4. Conclusions

Ferrocene- and ruthenocene-containing iridium(III) heteroleptic complexes of the type [(ppy)_2_Ir(RCOCHCOR′)] may easily be synthesized in moderate (30–70%) yields. These complexes are stable up to 200 ^o^C and may be stored for long times in air. A single crystal structure determination of **3** showed that ppy coordination is such that the pyridine fragments are *trans* relative to each other giving rise to a N-Ir-N angle approaching 180^o^. UV/vis spectroscopy shows complexes to absorb strongly in the UV region. Two weaker absorption bands are found in the visible light region between ca. 350 and 530 nm. The sequence of redox events is in the order Fc oxidation, then Ir^III^ oxidation and finally ruthenocene oxidation, all in one-electron transfer steps. The ferrocenyl, Ir^III^ and ruthenocenyl centres are all electrochemically reversibly oxidized, but generation of the ruthenocenium cation, Rc^+^, leads to compound degradation and loss of chemical reversibility of the Fc^+^/Fc and Ir^III/IV^ redox couples. The precise potential at which Ir^III^ are oxidized is dependent on the sum of Gordy scale group electronegativities, Σχ_R,_ of R groups of the β-diketonato ligand. DFT calculations showed that the energy of the HOMOs of the species involved in the Ir^III/IV^ oxidation, are inverse proportional to the redox potential of the Ir^III/IV^ couple. This relationship provides a linear correlation with 98 % accurate prediction of the redox potential of the Ir^III/IV^ couple of complexes of the type [(ppy)_2_Ir(RCOCHCORʹ)]. While [(ppy)_2_Ir(H_3_CCOCHCOCH_3_)] (**6**) exhibits phosphorescence with emission λ_max_ = 518 nm at 25 ^o^C, the ferrocenyl and ruthenocenyl molecular fragments of **1**–**5** quench all phosphorescence. Electrochemical generation of the oxidized species **6^+^** by generation of an Ir^IV^ moiety also cause phosphorescence quenching. Spectroelectrochemical results showed that Ir^IV^ generation led to very weak absorption patterns above 430 nm. These complexes may have potential as new antineoplastic drugs and they may also act as precursors towards iridium oxide and ruthenium oxide nano-electrocatalysts in electrochemical water splitting reactions. 

## Figures and Tables

**Table 1:** Crystal Data and Structural Refinement for **3**.

empirical formula	C_36.33_H_29.66_Cl_0.66_IrN_2_O_2_Ru	absorption coeff. (mm^−1^)	4.893
molecular weight	843.12	θ range for data collection (deg)	2.304–25.349
crystal size (mm^3^)	0.348 × 0.334 × 0.058	index ranges	−17 ≤ h ≤ 17
temperature (K)	150(2)		−11 ≤ k ≤ 11
wavelength (Å)	0.71073		−26 ≤ l ≤ 26
crystal system	monoclinic	no. of reflections collected	65183
space group	P2_1_/n	no. of independent reflections	5368
unit cell dim. (Å; deg)	*a* = 14.5533(18); *α* = 90	completeness to θ = 25.00°	96.1%
	*b* = 9.6595(12); *β* = 95.707(4)	refinement method	full-matrix least-squares on F^2^
	*c* = 22.053(3); *γ* = 90	data/restraints/parameters	5368/0/408
volume (Å^3^)	3084.8(7)	goodness of fit on *F*^2^	1.168
Z	4	final *R* indices [*I* > 2σ(I)]	R1 = 0.0500; wR2 = 0.1189
density (calc.) (g cm^−3^)	1.815	*R* indices (all data)	R1 = 0.0554; wR2 = 0.1219
*F*(000)	1640	largest diff. peak and hole (e Å^−3^)	1.58 and −0.93

**Table 2:** Wavelengths of absorption maxima (λ_max_) and extinction coefficients in brackets at these wavelengths, ε, for [(ppy)_2_Ir(RCOCHCORʹ)] complexes **1**–**6** in CH_2_Cl_2_ and Gordy scale group electronegativities, χ_R_, for R and Rʹ groups.

Complex (χ_R_)	λ_max_/nm (ε/M^−1^ cm^−1^)	Complex (χ_R_)	λ_max_/nm (ε/M^−1^ cm^−1^)
1: R = Fc (χ_Fc_ = 1.87) R′ = CH_3_ (χ_CH3_ = 2.34)	262 (38016); 346 (11374); 372 (sh,^b^ 9010); 457 (3575)	4: R = Rc (χ_Rc_ = 1.99), R′ = Rc (χ_Rc+_ = 2.87)	260 (42334); 350 (14083); 390 (14390); 462 (2721)
2: R = Fc (χ_Fc_ = 1.87), R′ = Fc (χ_Fc+_ = 2.82)^a^	256 (41428); 350 (11589); 384 (13205); 462 (5520)	5: R = Fc (χ_Fc_ = 1.87), ^a^ R′ = Rc (χ_(Rc)_: 1.89)	258 (40825); 351 (12518); 386 (13375); 465 (4076)
3: R = Rc χ_Rc_ = 1.99), R′ = CH_3_ (χ_CH3_ = 2.34)	262 (37568); 346 (11938); 371 (sh,^b^ 9010); 462 (2548)	6: R = R′ = CH_3_ (χ_CH3_ = 2.34)	260 (40897); 339 (9358); 405 (3979); 456 (2778)

^a^ (χ_Fc+_ = 2.82). ^b^ sh = shoulder.

**Table 3:** Cyclic voltammetry data of 0.5 mmol·dm^−3^ solutions of 1–5 in CH_2_Cl_2_ containing 0.1 mol·dm^−3^ [(^n^Bu)_4_N][B(C_6_F_5_)_4_] as supporting electrolyte at 20 ^°^C. Scan rate = 100 mV·s^−1^, potentials are vs. FcH/FcH^+^.

Wave	E_pa_/V	ΔE_p_/V	E^o′^/V	*i*_pa_/μA	i_pc_/i_pa_	Wave	E_pa_/V	ΔE_p_/V	E^o^′/V	*i*_pa_/μA	i_pc_/i_pa_
Decamethylferrocene, Fc*											
Fc*	−0.570	0.077	−0.608	1.80	1.00	**4, [(ppy)_2_Ir(RcCOCHCORc)]**					
						Ir^III/IV^	0.296	0.088	0.252	5.56	0.20
**1, [(ppy)_2_Ir(FcCOCHCOCH_3_)]**						R1	0.771	0.067	0.737	5.74	0.13
F1	0.126	0.081	0.086	6.03	1.00	R2_a_	0.984	0.181	0.893	5.56	0.30
Ir^III/IV^	0.539	0.085	0.497	5.69	0.97	R2_b_	1.179	- *^a^*	- *^a^*	0.37	- *^a^*
											
**2, [(ppy)_2_Ir(FcCOCHCOFc)]**						**5, [(ppy)_2_Ir(FcCOCHCORc)]**					
F1	0.090	0.080	0.050	5.96	0.94	F1	0.114	0.082	0.073	5.80	0.55
F2	0.277	0.083	0.235	5.58	1.00	Ir^III/IV^	0.487	0.083	0.445	5.40	0.78
Ir^III/IV^	0.721	0.082	0.681	5.77	1.00	R1	1.013	0.108	0.959	5.80	0.76
**3, [(ppy)_2_Ir(RcCOCHCOCH_3_)]**						**6, [(ppy)_2_Ir(CH_3_COCHCOCH_3_]**					
Ir^III/IV^	0.327	0.087	0.283	5.68	0.32	Ir^III/IV^	0.359	0.081	0.318	5.94	1.00
R1_a_	0.889	0.083	0.847	6.91	0.04						
R1_b_	0.951	- *^a^*	- *^a^*	2.76	- *^a^*						

*^a^* Corresponding cathodic signal could not be assigned.

## Data Availability

CCDC 1916701 contains the supplementary crystallographic data for COMPLEX **3** of this paper. These data can be obtained free of charge via http://www.ccdc.cam.ac.uk/conts/retrieving.html (or from the CCDC, 12 Union Road, Cambridge CB2 1EZ, UK; Fax: +44 1223 336033; E-mail: deposit@ccdc.cam.ac.uk).

## Supplementary Materials

^1^H-NMR Spectra of **1**–**5**; IR Spectra **1**–**5**; Electrochemical Schemes for **1**–**6**; Crystallographic C-H···O interactions within **3**; Tables providing crystallographic information; DFT figures, data and optimized coordinates in the gas phase as well as in DCM.
