# Supplementary material for: Synthesis, Spectroscopy and Electrochemistry in Relation to DFT Computed Energies of Ferrocene- and Ruthenocene-Containing β-Diketonato Iridium(III) Heteroleptic Complexes. Structure of [(2-Pyridylphenyl)2Ir(RcCOCHCOCH3]
Source: Molecules. 2019 Oct 30;24(21):3923. doi: 10.3390/molecules24213923 (PMC6864483; doi:10.3390/molecules24213923)
Supplement: Supplementary file 1 [file molecules-24-03923-s001.pdf]

Supplementary Information for:

Synthesis, spectroscopy and electrochemistry in relation to ADF computed energies of ferrocene- and ruthenocene-containing β-diketonato iridium(III) heteroleptic complexes. Structure of [(2-phenylpyridine)$_2$Ir(RcCOCHCOCH$_3$)]†

Blenerhassitt E. Buitendach, Jeanet Conradie, Frederick P. Malan, J. W. (Hans) Niemantsverdriet and Jannie C. Swarts

Contents

A. ^{1}H NMR spectrums of complexes 1 – 5 2
B. FT-IR spectrums of complexes 1 – 5. 5
C. Electrochemical Schemes for 1 – 6. 8
D. Crystallographic C-H···O interactions within 3. 10
E. Crystallographic Information of 3 11
F. DFT Figures. 23
G. DFT data. 24
H. DFT Optimized Coordinates. 24
1$^+$, [(ppy)$_2$Ir^{III}(Fc$^+$COCHCOCH$_3$)] gas phase 24
2^{2+}, [(ppy)$_2$Ir^{III}(Fc$^+$COCHCOFc$^+$)] gas phase 25
3, [(ppy)$_2$Ir^{III}(RuCOCHCOCH$_3$)] gas phase 27
4, [(ppy)$_2$Ir^{III}(RuCOCHCORu)] gas phase 29
5$^+$, [(ppy)$_2$Ir^{III}(Fc$^+$COCHCORu)] gas phase 30
6, [(ppy)$_2$Ir^{III}(CH$_3$COCHCOCH$_3$)] gas phase 32
1$^+$, [(ppy)$_2$Ir^{III}(Fc$^+$COCHCOCH$_3$)] DCM 33
2^{2+}, [(ppy)$_2$Ir^{III}(Fc$^+$COCHCOFc$^+$)] DCM 35
3, [(ppy)$_2$Ir^{III}(RuCOCHCOCH$_3$)] DCM 37
4, [(ppy)$_2$Ir^{III}(RuCOCHCORu)] DCM 38
5$^+$, [(ppy)$_2$Ir^{III}(Fc$^+$COCHCORu)] DCM 40
6, [(ppy)$_2$Ir^{III}(CH$_3$COCHCOCH$_3$)] DCM 42

A. ¹H NMR spectrums of complexes 1 – 5.

1 – Ir(ppy)$_2$(FcCOCHCOCH$_3$)

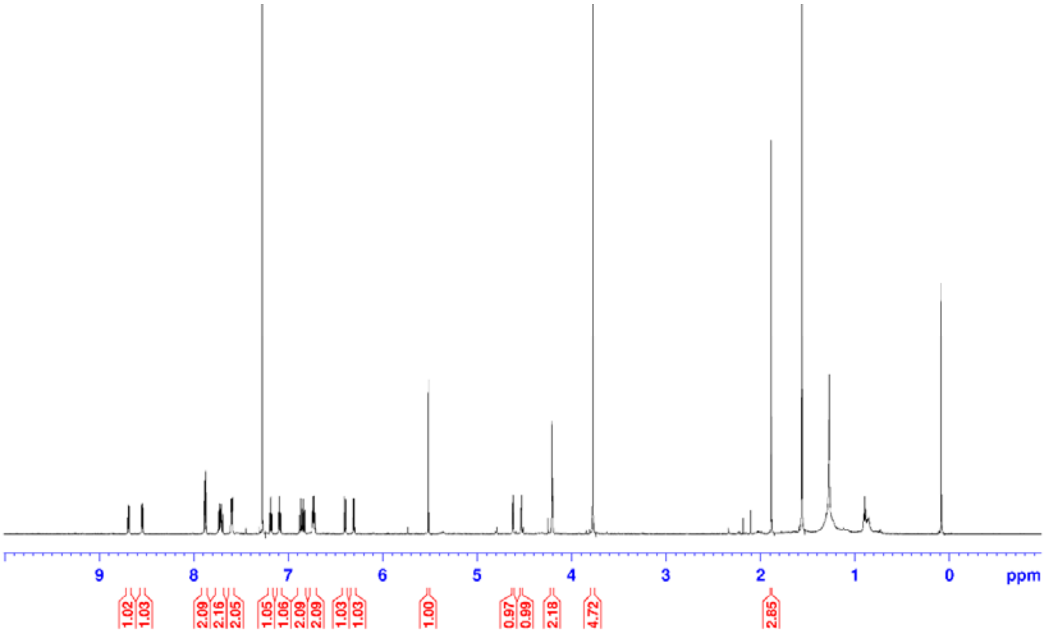

2 – Ir(ppy)$_2$(FcCOCHCOFc)

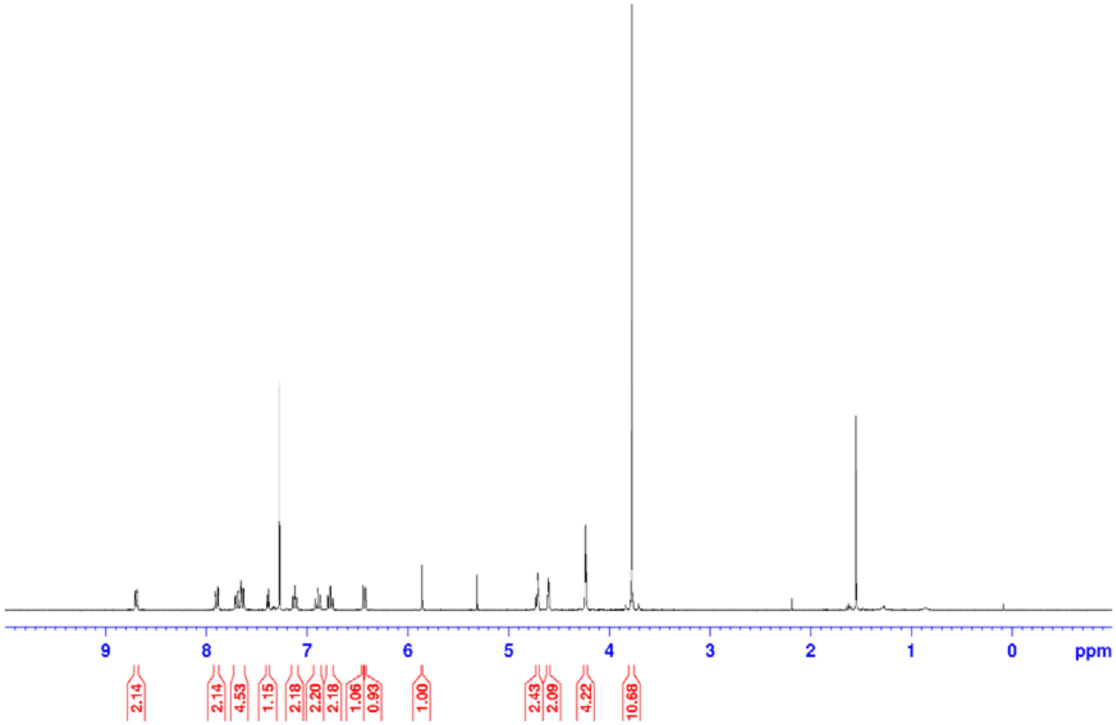

3 – Ir(ppy)$_2$(RcCOCHCOCH$_3$)

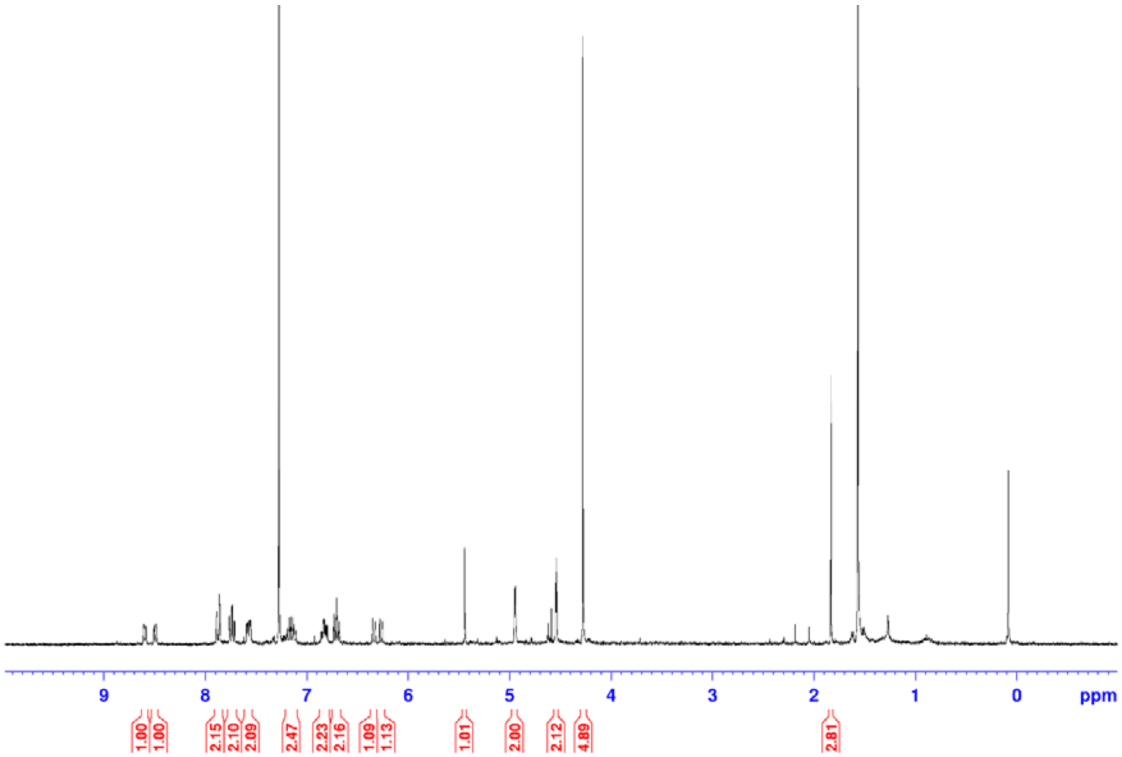

4 – Ir(ppy)$_2$(RcCOCHCORc)

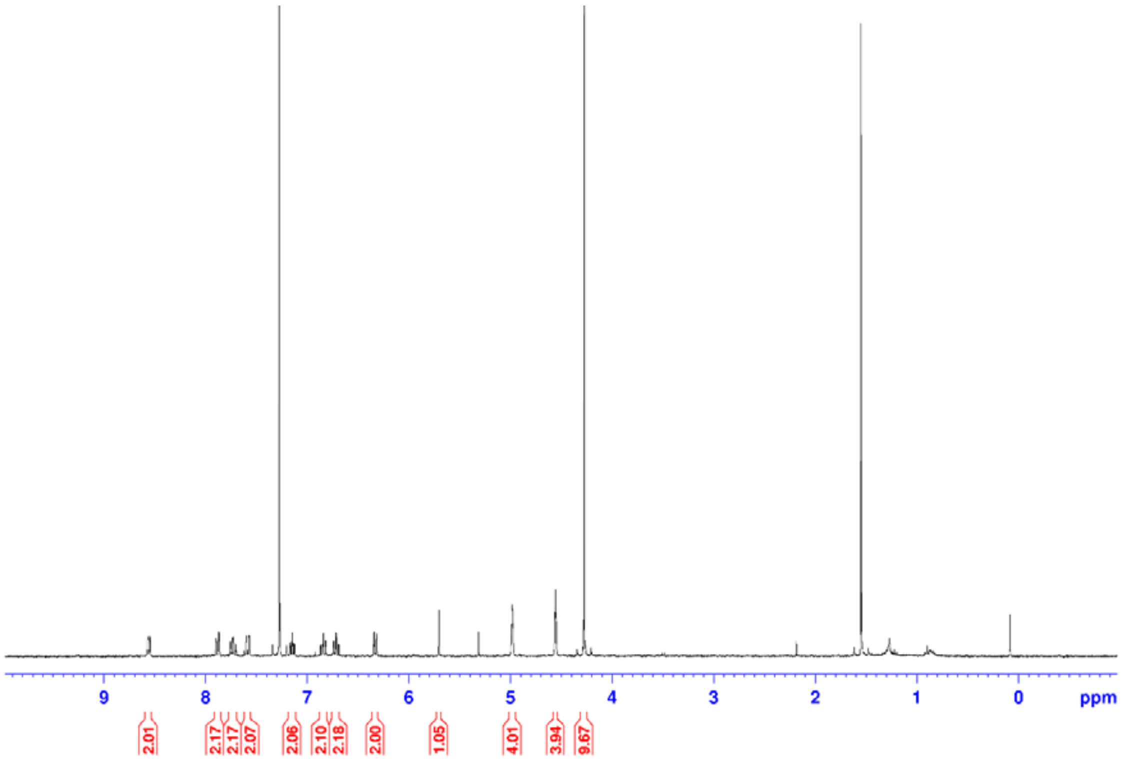

5 – Ir(ppy)$_2$(FcCOCHCORc)

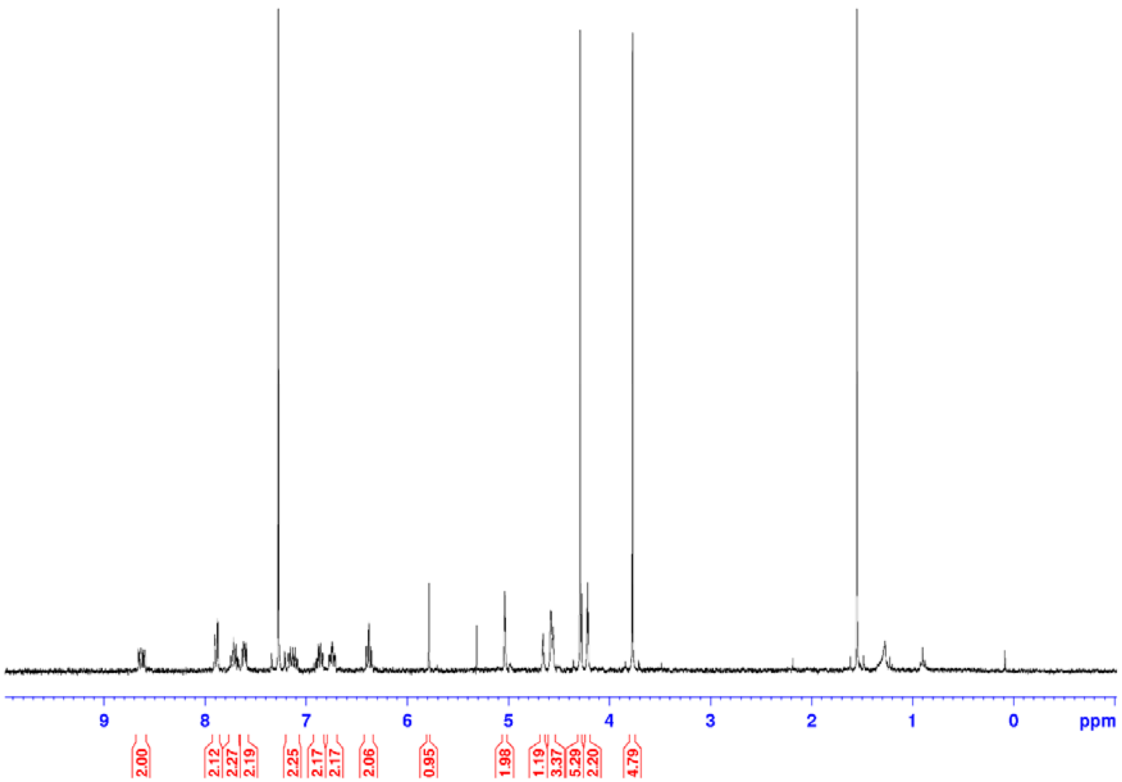

B. FT-IR spectrums of complexes 1 – 5.

1 – Ir(ppy)$_2$(FcCOCHCOCH$_3$)

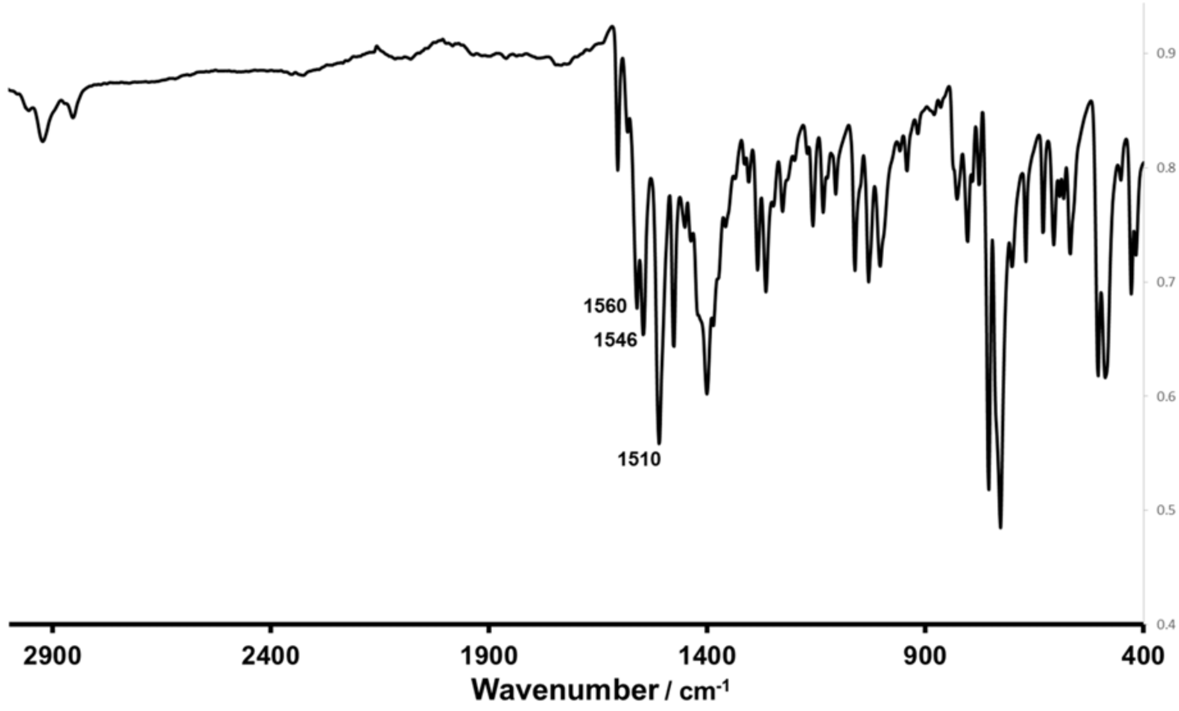

2 – Ir(ppy)$_2$(FcCOCHCOFc)

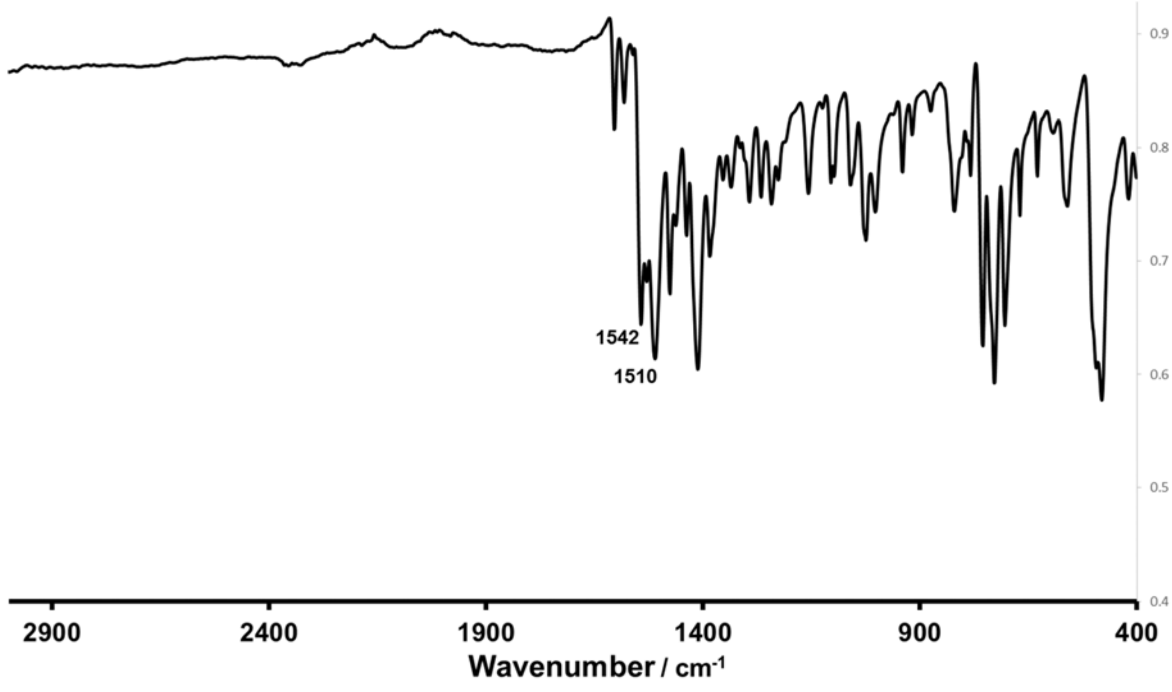

3 – Ir(ppy)$_2$(RcCOCHCOCH$_3$)

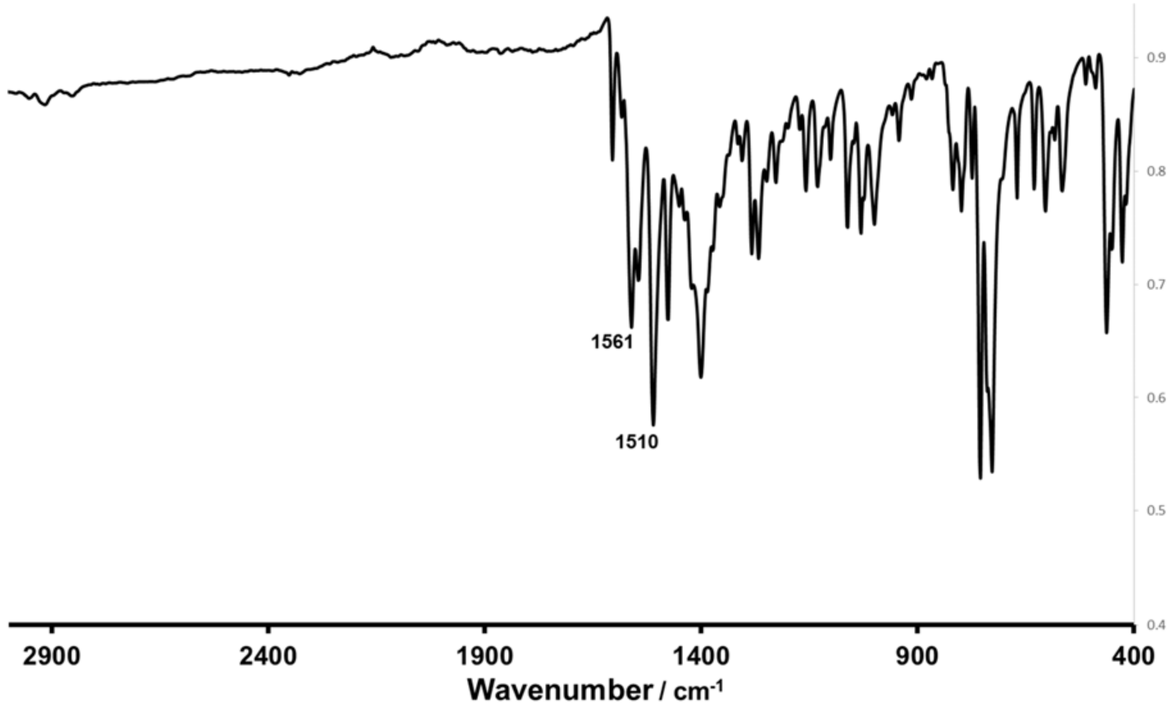

4 – Ir(ppy)$_2$(RcCOCHCORc)

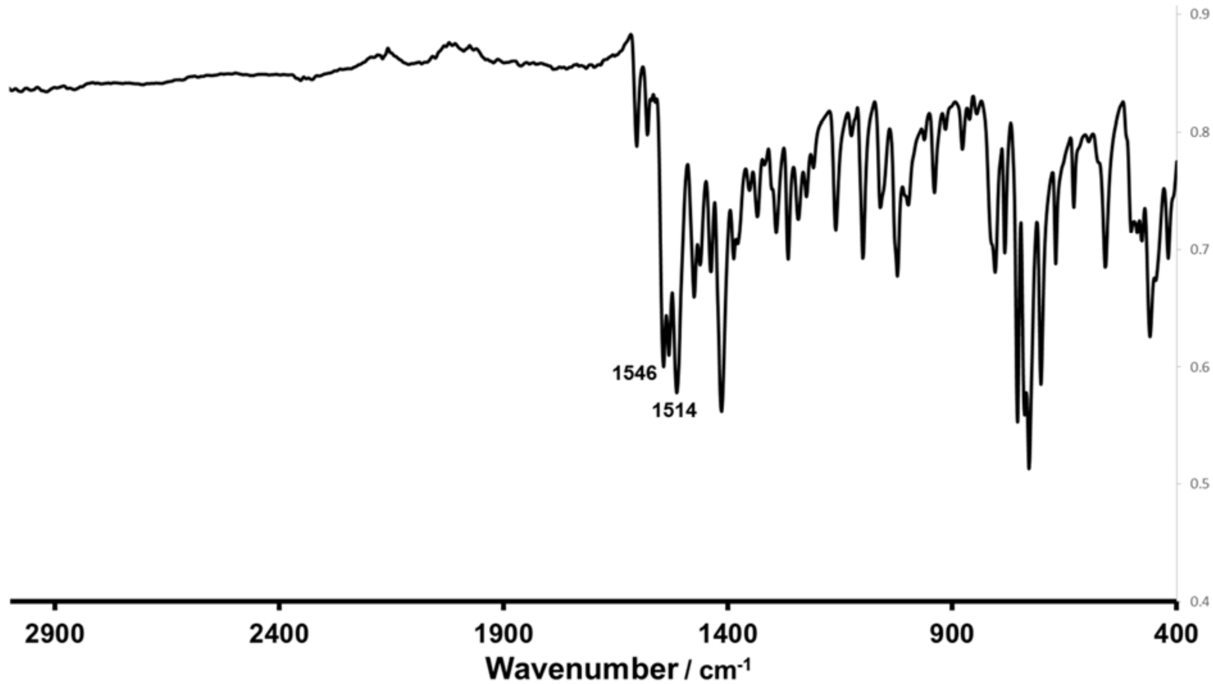

5 – Ir(ppy)$_2$(FcCOCHCORc)

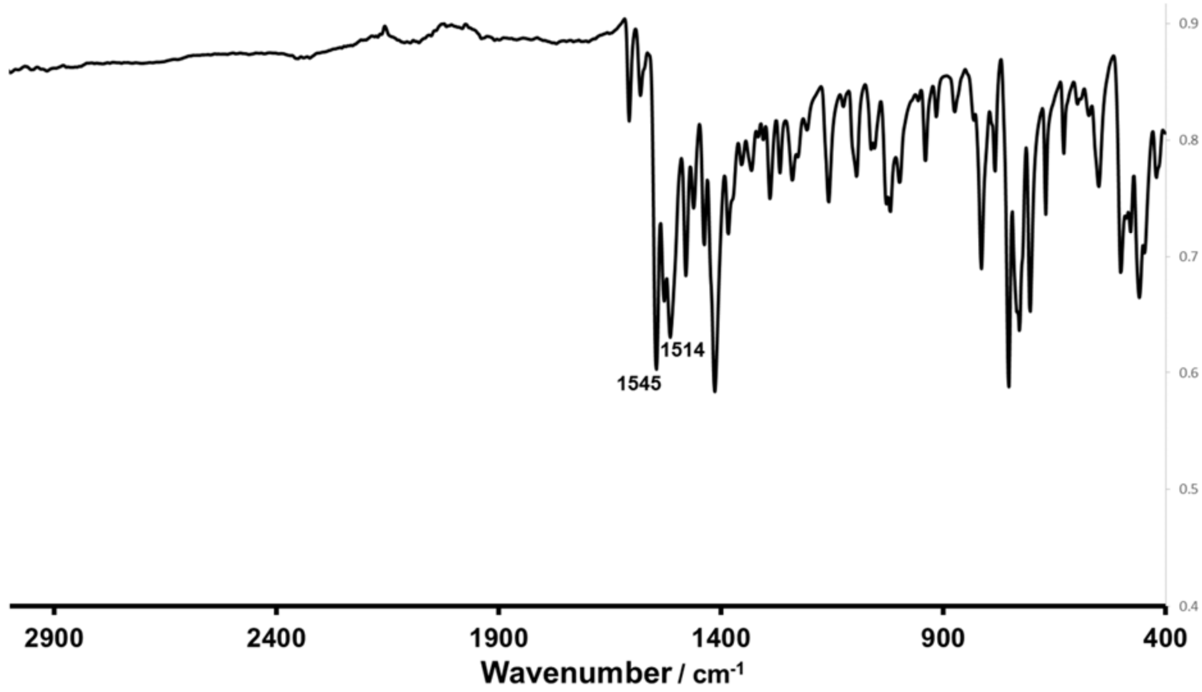

C. Electrochemical Schemes for 1 – 6.

1, $[(ppy)_2Ir(FcCOCHCOCH_3)]$

$[(ppy)_2Ir^{III}(FcCOCHCOCH_3)]$ **(ground state)**

Wave F1 $E^{o'} = 0.086$ V

+ $1e^-$ ⇅ - $1e^-$

$[(ppy)_2Ir^{III}(Fc^+COCHCOCH_3)]^+$

Wave Ir $E^{o'} = 0.497$ V

+ $1e^-$ ⇅ - $1e^-$

$[(ppy)_2Ir^{IV}(Fc^+COCHCOCH_3)]^{2+}$

2, $[(ppy)_2Ir(FcCOCHCOFc)]$

$[(ppy)_2Ir^{III}(FcCOCHCOFc)]$ **(ground state)**

Wave F1 $E^{o'} = 0.050$ V

+ $1e^-$ ⇅ - $1e^-$

$[(ppy)_2Ir^{III}(Fc^+COCHCOFc)]^+$

Wave F2 $E^{o'} = 0.235$ V

+ $1e^-$ ⇅ - $1e^-$

$[(ppy)_2Ir^{III}(Fc^+COCHCOFc^+)]^{2+}$

Wave Ir $E^{o'} = 0.681$ V

+ $1e^-$ ⇅ - $1e^-$

$[(ppy)_2Ir^{IV}(Fc^+COCHCOFc^+)]^{3+}$

3, $[(ppy)_2Ir(RcCOCHCOCH_3)]$

$[(ppy)_2Ir^{III}(RcCOCHCOCH_3)]$ **(ground state)**

Wave Ir $E^{o'} = 0.283$ V

+ $1e^-$ ⇅ - $1e^-$

$[(ppy)_2Ir^{IV}(RcCOCHCOCH_3)]^+$

Wave R1 $E^{o'} = 0.847$ V

+ $1e^-$ ⇅ - $1e^-$

$[(ppy)_2Ir^{IV}(Rc^+COCHCOCH_3)]^{2+}$

4, $[(ppy)_2Ir(RcCOCHCORc)]$

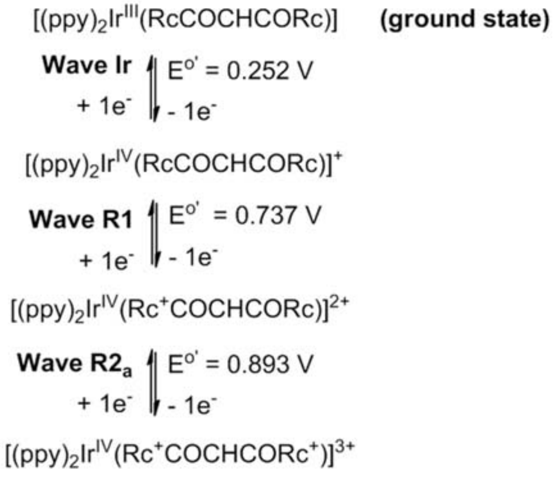

5, $[(ppy)_2Ir(FcCOCHCORc)]$

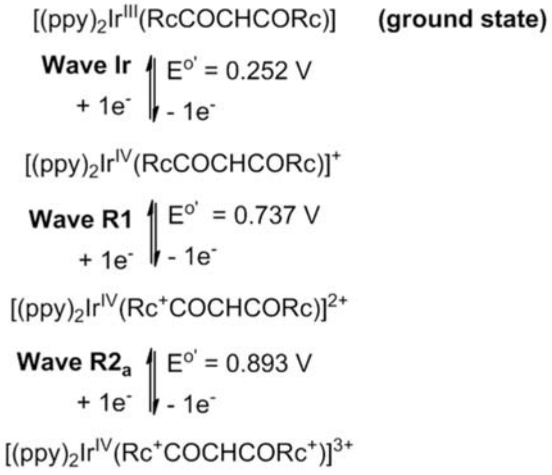

6, $[(ppy)_2Ir(CH_3COCHCOCH_3]$

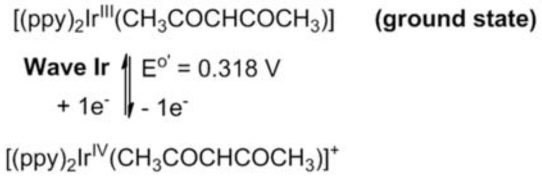

D. Crystallographic C-H···O interactions within 3.

Each molecule contains two intramolecular, and one intermolecular C-H···O interaction (Figure S1). The two intramolecular C-H···O interactions exist between the atoms C15-H15···O1 (H···O = 2.50 Å) and C26-H26···O2 (H···O = 2.56 Å). They 'lock' the two *cis*- pyridylphenyl ligands in molecular place with slight octahedral distortion of the iridium center. The bisecting axial Ir core bonds showed deviations from linearity with bond angles of N(1)–Ir(1)–N(2) = 174.3(3)°, O(1)–Ir(1)–C(25) = 172.9(3)° and O(2)–Ir(1)–C(36) = 172.3(3)°. This is a slight but noticeable octahedral distortion of the Ir core. The intermolecular C-H···O interaction is a conventional interaction of $(C11\text{-}H11)_{\text{molecule A}}\cdots(O1)_{\text{molecule B}}$ (2.63 Å) (H11 is a proton from one of the Cp rings of the ruthenocenyl moiety). Collectively, these C-H···O interactions form part of a complex three-dimensional network and enhanced stabilization of the overall structure.

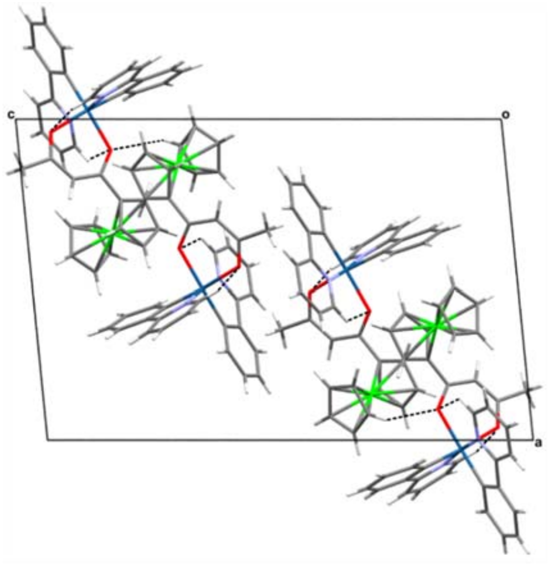

Figure S1. Extended network of C–H···O interactions within **3**, viewed down the b axis. C–H···O interactions are indicated by black dashed lines.

E. Crystallographic Information of 3

Table S1 Fractional Atomic Coordinates (×10^4) and Equivalent Isotropic Displacement Parameters (Å^2×10^3) for 3. Ueq is defined as 1/3 of the trace of the orthogonalised U_{IJ} tensor.

Atom	x	y	z	U(eq) (Å^2)
Ir1	5288.2(2)	2529.6(4)	6498.9(2)	32.25(12)
Ru1	1432.6(5)	3765.4(9)	6762.2(4)	43.6(2)
O1	4029(4)	2399(7)	6897(3)	39.5(15)
N1	4970(5)	4433(8)	6129(3)	32.0(16)
C1	3273(7)	1949(10)	6642(4)	43(2)
O2	4599(4)	1555(7)	5699(3)	36.7(14)
N2	5742(5)	688(8)	6876(3)	33.0(16)
C2	3132(7)	1311(11)	6069(5)	47(2)
C3	3777(6)	1159(10)	5647(4)	40(2)
C4	3460(8)	463(11)	5040(5)	49(3)
C5	2467(6)	2184(10)	7003(5)	41(2)
C6	2471(7)	3169(12)	7488(5)	47(2)
C7	1585(8)	3153(13)	7716(5)	55(3)
C8	1047(8)	2154(12)	7382(5)	56(3)
C9	1585(7)	1579(11)	6935(5)	52(3)
C10	1690(8)	5809(12)	6403(5)	52(3)
C11	821(7)	5818(12)	6653(5)	54(3)
C12	243(7)	4853(13)	6324(5)	59(3)
C13	746(8)	4241(14)	5868(5)	59(3)
C14	1628(7)	4847(12)	5927(4)	49(3)

C15	4167(7)	5100(10)	6167(4)	42(2)
C16	3972(7)	6354(11)	5869(4)	46(2)
C17	4606(7)	6912(11)	5518(4)	44(2)
C18	5439(7)	6250(10)	5495(4)	41(2)
C19	5625(6)	5006(10)	5804(4)	37(2)
C20	6460(6)	4196(10)	5808(4)	34(2)
C21	7226(7)	4613(11)	5522(4)	45(2)
C22	7983(6)	3762(12)	5520(4)	44(2)
C23	7980(7)	2484(12)	5804(4)	48(2)
C24	7225(6)	2060(11)	6098(4)	38(2)
C25	6443(6)	2903(9)	6112(4)	32.8(19)
C26	5670(6)	-550(10)	6600(4)	35(2)
C27	6045(7)	-1722(10)	6855(4)	41(2)
C28	6552(7)	-1640(10)	7421(4)	41(2)
C29	6632(6)	-389(10)	7714(4)	36(2)
C30	6219(6)	786(10)	7448(4)	34(2)
C31	6230(6)	2171(10)	7701(4)	36(2)
C32	6554(6)	2475(11)	8306(4)	41(2)
C33	6494(7)	3815(12)	8513(5)	49(3)
C34	6129(7)	4838(11)	8134(5)	47(2)
C35	5836(7)	4549(10)	7530(4)	43(2)
C36	5858(6)	3217(10)	7299(4)	35(2)
C37	9720(40)	1710(60)	4480(30)	106(18)
Cl1	10580(20)	1020(40)	4993(13)	255(18)
Cl2	8860(30)	970(40)	4408(16)	320(30)

Table S2 Hydrogen Atom Positions and Isotropic Displacement Parameters

Atom	x	y	z	U(iso) (Å^2)
H2	2533.87	943.6	5956.71	57
H4A	3295.71	1173.12	4730.56	74
H4B	2919.4	-117.85	5087.7	74
H4C	3960.22	-112.66	4912.1	74
H6	3011.13	3744	7651.31	57
H7	1396.81	3707.11	8066.14	66
H8	401.95	1880.15	7449.55	67
H9	1380.75	824.53	6641.49	62
H10	2231.19	6417.7	6530.53	62
H11	644.79	6431.24	6987.97	64
H12	-417.02	4662.34	6385.35	70
H13	503.6	3558.85	5550.23	71
H14	2127.81	4647.31	5659.11	59
H15	3721.38	4707.06	6402.58	51
H16	3405.07	6817.35	5907.91	55
H17	4472.61	7741.18	5294.68	53
H18	5892.94	6644.73	5265.87	49
H21	7225.64	5490.17	5327.34	53
H22	8503.71	4048.28	5324.21	53
H23	8499.4	1890.33	5797.93	58
H24	7240	1182.19	6293.35	46
H26	5340.05	-607.86	6207.13	42
H27	5961.5	-2585.1	6650.41	49

H28	6840.21	-2441.6	7603.13	49
H29	6974.22	-325.69	8102.88	43
H32	6811.66	1767.7	8569.49	50
H33	6708.34	4029.42	8923.23	59
H34	6076.12	5752.74	8285.32	57
H35	5614.1	5281.98	7268.21	51
H37A	9589.4	2669.13	4605.44	127
H37B	9956.77	1771.29	4072.06	127

Table S3 Anisotropic Displacement Parameters

Atom	U(1,1) or U	U(2,2)	U(3,3)	U(2,3)	U(1,3)	U(1,2)
Ir1	32.86(19)	33.2(2)	30.09(19)	1.12(16)	0.02(13)	-0.23(15)
Ru1	33.4(4)	54.3(5)	43.4(4)	1.5(4)	5.4(3)	0.5(4)
O1	34(3)	47(4)	38(3)	2(3)	3(3)	0(3)
N1	33(4)	35(4)	27(4)	-2(3)	-2(3)	1(3)
C1	45(6)	36(5)	46(6)	8(5)	0(4)	0(4)
O2	36(3)	41(4)	32(3)	1(3)	-3(3)	0(3)
N2	33(4)	36(4)	31(4)	9(3)	6(3)	-3(3)
C2	38(5)	52(6)	49(6)	-6(5)	-6(4)	-10(5)
C3	41(5)	38(5)	38(5)	1(4)	-8(4)	2(4)
C4	59(6)	44(6)	44(6)	-2(5)	-5(5)	-6(5)
C5	35(5)	38(5)	50(6)	6(4)	1(4)	-1(4)
C6	42(5)	57(7)	42(5)	1(5)	5(4)	-2(5)
C7	52(6)	67(7)	48(6)	6(6)	9(5)	10(6)
C8	43(6)	59(7)	67(7)	15(6)	17(5)	0(5)

C9	56(6)	37(6)	63(7)	-1(5)	8(5)	-12(5)
C10	54(6)	51(7)	50(6)	17(5)	1(5)	4(5)
C11	46(6)	56(7)	57(6)	5(6)	2(5)	13(5)
C12	41(6)	72(8)	61(7)	7(6)	-4(5)	6(6)
C13	52(6)	80(9)	43(6)	-2(6)	-8(5)	0(6)
C14	50(6)	59(7)	38(5)	7(5)	5(4)	3(5)
C15	45(5)	36(5)	44(5)	1(4)	-4(4)	3(4)
C16	45(5)	46(6)	46(6)	-4(5)	0(4)	7(5)
C17	57(6)	35(5)	37(5)	1(4)	-5(4)	4(5)
C18	55(6)	35(5)	32(5)	3(4)	-1(4)	-8(5)
C19	43(5)	36(5)	31(4)	-2(4)	-4(4)	-11(4)
C20	33(5)	40(5)	27(4)	-4(4)	-4(3)	-9(4)
C21	52(6)	47(6)	32(5)	6(4)	-2(4)	-6(5)
C22	33(5)	63(7)	37(5)	11(5)	5(4)	-10(5)
C23	43(5)	60(7)	40(5)	-5(5)	-1(4)	4(5)
C24	41(5)	44(5)	30(5)	5(4)	1(4)	1(4)
C25	33(4)	39(5)	26(4)	-6(4)	0(3)	-3(4)
C26	25(4)	45(5)	35(5)	-4(4)	2(3)	3(4)
C27	45(5)	30(5)	47(6)	0(4)	5(4)	-3(4)
C28	45(5)	36(5)	42(5)	8(4)	6(4)	2(4)
C29	26(4)	46(6)	36(5)	8(4)	-2(4)	-2(4)
C30	24(4)	44(5)	34(5)	1(4)	6(3)	-2(4)
C31	32(5)	40(5)	35(5)	2(4)	0(4)	-4(4)
C32	39(5)	46(6)	38(5)	2(5)	0(4)	-2(4)
C33	53(6)	56(7)	36(5)	-6(5)	-6(4)	-9(5)

C34	56(6)	38(6)	46(6)	-1(5)	-3(5)	-3(5)
C35	51(6)	37(5)	38(5)	0(4)	-6(4)	2(4)
C36	36(5)	37(5)	32(5)	-8(4)	-4(4)	-4(4)
C37	90(30)	110(40)	110(40)	-30(40)	0(30)	-30(30)
Cl1	290(40)	320(40)	160(20)	-40(30)	40(20)	120(30)
Cl2	420(50)	330(50)	250(30)	-170(40)	190(40)	-220(40)

Table S4 Bond Distances (Å)

Bond	Distance (Å)	Bond	Distance (Å)	Bond	Distance (Å)
Ir1-O1	2.112(6)	O2-C3	1.250(11)	C20-C21	1.394(13)
Ir1-N1	2.046(7)	N2-C26	1.341(12)	C20-C25	1.419(13)
Ir1-O2	2.158(6)	N2-C30	1.381(11)	C21-C22	1.374(14)
Ir1-N2	2.045(7)	C2-C3	1.393(14)	C22-C23	1.384(15)
Ir1-C25	1.992(9)	C3-C4	1.528(13)	C23-C24	1.392(14)
Ir1-C36	1.987(8)	C5-C6	1.431(15)	C24-C25	1.402(13)
Ru1-C5	2.173(9)	C5-C9	1.404(14)	C26-C27	1.355(13)
Ru1-C6	2.167(11)	C6-C7	1.430(15)	C27-C28	1.388(13)
Ru1-C7	2.175(11)	C7-C8	1.405(17)	C28-C29	1.370(13)
Ru1-C8	2.182(11)	C8-C9	1.431(15)	C29-C30	1.387(13)
Ru1-C9	2.154(11)	C10-C11	1.429(15)	C30-C31	1.449(13)
Ru1-C10	2.174(11)	C10-C14	1.399(15)	C31-C32	1.401(13)
Ru1-C11	2.177(11)	C11-C12	1.407(16)	C31-C36	1.416(13)
Ru1-C12	2.169(11)	C12-C13	1.430(16)	C32-C33	1.378(14)
Ru1-C13	2.170(11)	C13-C14	1.405(15)	C33-C34	1.368(14)
Ru1-C14	2.161(10)	C15-C16	1.393(14)	C34-C35	1.386(13)

N1-C15	1.344(12)	C16-C17	1.373(14)	C35-C36	1.386(13)
N1-C19	1.365(11)	C17-C18	1.376(14)	C37-Cl1	1.74(6)
C1-C2	1.402(14)	C18-C19	1.395(13)	C37-Cl2	1.43(5)
C1-C5	1.500(14)	C19-C20	1.445(13)	Cl1-Cl2[1]	2.42(6)

[1]2-X,-Y,1-Z

Table S5 Bond Angles (º)

Bonds	Angle (º)	Bonds	Angle (º)	Bonds	Angle (º)
O1-Ir1-O2	87.8(2)	O2-C3-C4	114.6(9)	C12-Ru1-C7	124.3(4)
N1-Ir1-O1	92.7(3)	C2-C3-C4	117.8(9)	C12-Ru1-C8	112.4(4)
N1-Ir1-O2	89.8(3)	C1-C5-Ru1	122.4(7)	C12-Ru1-C10	63.7(4)
N2-Ir1-O1	92.1(3)	C6-C5-Ru1	70.5(6)	C12-Ru1-C11	37.8(4)
N2-Ir1-N1	174.3(3)	C6-C5-C1	123.5(9)	C12-Ru1-C13	38.5(4)
N2-Ir1-O2	93.3(3)	C9-C5-Ru1	70.3(6)	C13-Ru1-C5	128.0(4)
C25-Ir1-O1	172.9(3)	C9-C5-C1	129.5(10)	C13-Ru1-C7	158.5(4)
C25-Ir1-N1	80.4(3)	C9-C5-C6	107.0(9)	C13-Ru1-C8	126.6(5)
C25-Ir1-O2	94.1(3)	C5-C6-Ru1	71.0(6)	C13-Ru1-C10	63.7(5)
C25-Ir1-N2	94.6(3)	C7-C6-Ru1	71.1(6)	C13-Ru1-C11	63.9(5)
C36-Ir1-O1	87.3(3)	C7-C6-C5	108.6(10)	C14-Ru1-C5	113.7(4)
C36-Ir1-N1	96.4(3)	C6-C7-Ru1	70.5(6)	C14-Ru1-C6	128.1(4)
C36-Ir1-O2	172.3(3)	C8-C7-Ru1	71.4(6)	C14-Ru1-C7	161.2(4)
C36-Ir1-N2	80.8(3)	C8-C7-C6	107.4(10)	C14-Ru1-C8	160.6(5)
C36-Ir1-C25	91.4(4)	C7-C8-Ru1	71.0(6)	C14-Ru1-C10	37.7(4)
C5-Ru1-C7	64.6(4)	C7-C8-C9	108.2(9)	C14-Ru1-C11	63.4(4)
C5-Ru1-C8	63.9(4)	C9-C8-Ru1	69.7(6)	C14-Ru1-C12	63.5(4)

C5-Ru1-C10	126.0(4)	C5-C9-Ru1	71.8(6)	C14-Ru1-C13	37.9(4)
C5-Ru1-C11	158.9(4)	C5-C9-C8	108.8(10)	C1-O1-Ir1	126.1(6)
C6-Ru1-C5	38.5(4)	C8-C9-Ru1	71.8(6)	C15-N1-Ir1	124.7(6)
C6-Ru1-C7	38.4(4)	C11-C10-Ru1	71.0(6)	C15-N1-C19	119.7(8)
C6-Ru1-C8	63.4(4)	C14-C10-Ru1	70.7(6)	C19-N1-Ir1	115.5(6)
C6-Ru1-C10	112.2(4)	C14-C10-C11	107.4(10)	O1-C1-C2	126.6(9)
C6-Ru1-C11	124.9(4)	C10-C11-Ru1	70.7(6)	O1-C1-C5	113.9(9)
C6-Ru1-C12	157.7(4)	C12-C11-Ru1	70.8(7)	C2-C1-C5	119.5(9)
C6-Ru1-C13	162.2(4)	C12-C11-C10	107.7(10)	C3-O2 Ir1	124.8(6)
C7-Ru1-C8	37.6(5)	C11-C12-Ru1	71.4(6)	C26-N2-Ir1	125.7(6)
C7-Ru1-C11	110.9(4)	C11-C12-C13	108.3(10)	C26-N2-C30	119.0(8)
C9-Ru1-C5	37.9(4)	C13-C12-Ru1	70.8(6)	C30-N2-Ir1	115.1(6)
C9-Ru1-C6	63.7(4)	C12-C13-Ru1	70.7(6)	C3-C2-C1	126.9(9)
C9-Ru1-C7	64.1(5)	C14-C13-Ru1	70.7(6)	O2-C3-C2	127.4(8)
C9-Ru1-C8	38.5(4)	C14-C13-C12	106.8(10)	C21-C20-C19	123.5(9)
C9-Ru1-C10	159.5(4)	C10-C14-Ru1	71.7(6)	C21-C20-C25	121.4(9)
C9-Ru1-C11	161.2(4)	C10-C14-C13	109.7(10)	C25-C20-C19	115.1(8)
C9-Ru1-C12	128.1(5)	C13-C14-Ru1	71.4(6)	C22-C21-C20	120.4(9)
C9-Ru1-C13	113.5(5)	N1-C15-C16	121.7(9)	C21-C22-C23	119.6(9)
C9-Ru1-C14	127.2(4)	C17-C16-C15	119.4(10)	C22-C23-C24	120.8(10)
C10-Ru1-C7	126.5(5)	C16-C17-C18	118.9(9)	C23-C24-C25	121.3(9)
C10-Ru1-C8	160.2(4)	C17-C18-C19	120.7(9)	C20-C25-Ir1	114.7(6)
C10-Ru1-C11	38.4(4)	N1-C19-C18	119.6(9)	C24-C25-Ir1	128.7(7)
C11-Ru1-C8	126.2(4)	N1-C19-C20	114.1(8)	C24-C25-C20	116.6(8)
C12-Ru1-C5	162.2(4)	C18-C19-C20	126.2(9)	N2-C26-C27	123.2(8)

C26-C27-C28	118.8(9)	C32-C31-C30	123.4(9)	C36-C35-C34	121.7(9)
C29-C28-C27	119.1(9)	C32-C31-C36	121.2(9)	C31-C36-Ir1	114.7(6)
C28-C29-C30	120.6(8)	C36-C31-C30	115.4(8)	C35-C36-Ir1	127.9(7)
N2-C30-C29	119.2(8)	C33-C32-C31	119.1(9)	C35-C36-C31	116.9(8)
N2-C30-C31	113.6(8)	C34-C33-C32	120.7(9)	Cl2-C37-Cl1	116(5)
C29-C30-C31	127.2(8)	C33-C34-C35	120.3(10)	C37-Cl1-Cl2[1]	147(3)
C37-Cl2-Cl1[1]	96(4)				

[1]2-X,-Y,1-Z

Table S6 Torsion Angles (º)

Bonds	Angle (º)	Bonds	Angle (º)
Ir1-O1-C1-C2	7.2(14)	C10-C11-C12-C13	-0.1(13)
Ir1-O1-C1-C5	-171.1(6)	C11-C10-C14-Ru1	-61.8(7)
Ir1-N1-C15-C16	-175.6(7)	C11-C10-C14-C13	-0.2(12)
Ir1-N1-C19-C18	174.9(6)	C11-C12-C13-Ru1	61.9(8)
Ir1-N1-C19-C20	-3.4(9)	C11-C12-C13-C14	0.0(13)
Ir1-O2-C3-C2	3.1(14)	C12-C13-C14-Ru1	61.9(8)
Ir1-O2-C3-C4	-178.4(6)	C12-C13-C14-C10	0.1(13)
Ir1-N2-C26-C27	174.8(7)	C14-C10-C11-Ru1	61.6(7)
Ir1-N2-C30-C29	-173.6(6)	C14-C10-C11-C12	0.2(12)
Ir1-N2-C30-C31	5.8(9)	C15-N1-C19-C18	-2.6(12)
Ru1-C5-C6-C7	61.5(8)	C15-N1-C19-C20	179.1(8)
Ru1-C5-C9-C8	-62.7(8)	C15-C16-C17-C18	-3.2(14)
Ru1-C6-C7-C8	62.3(8)	C16-C17-C18-C19	2.3(14)
Ru1-C7-C8-C9	60.0(8)	C17-C18-C19-N1	0.6(13)

Ru1-C8-C9-C5	62.7(7)	C17-C18-C19-C20	178.7(9)
Ru1-C10-C11-C12	-61.4(8)	C18-C19-C20-C21	3.9(14)
Ru1-C10-C14-C13	61.7(8)	C18-C19-C20-C25	-174.3(8)
Ru1-C11-C12-C13	-61.5(8)	C19-N1-C15-C16	1.7(13)
Ru1-C12-C13-C14	-61.9(8)	C19-C20-C21-C22	-177.0(9)
Ru1-C13-C14-C10	-61.8(8)	C19-C20-C25-Ir1	-2.6(9)
O1-C1-C2-C3	-6.1(18)	C19-C20-C25-C24	176.9(8)
O1-C1-C5-Ru1	105.1(9)	C20-C21-C22-C23	0.0(14)
O1-C1-C5-C6	18.2(14)	C21-C20-C25-Ir1	179.1(7)
O1-C1-C5-C9	-164.5(10)	C21-C20-C25-C24	-1.3(12)
N1-C15-C16-C17	1.2(14)	C21-C22-C23-C24	-0.8(15)
N1-C19-C20-C21	-177.9(8)	C22-C23-C24-C25	0.6(14)
N1-C19-C20-C25	3.9(11)	C23-C24-C25-Ir1	180.0(7)
C1-C2-C3-O2	0.3(18)	C23-C24-C25-C20	0.5(13)
C1-C2-C3-C4	-178.2(10)	C25-C20-C21-C22	1.1(14)
C1-C5-C6-Ru1	116.6(9)	C26-N2-C30-C29	1.8(12)
C1-C5-C6-C7	178.1(9)	C26-N2-C30-C31	-178.8(7)
C1-C5-C9-Ru1	-116.3(10)	C26-C27-C28-C29	2.2(14)
C1-C5-C9-C8	-178.9(10)	C27-C28-C29-C30	-0.5(14)
N2-C26-C27-C28	-2.0(14)	C28-C29-C30-N2	-1.5(13)
N2-C30-C31-C32	170.4(8)	C28-C29-C30-C31	179.2(9)
N2-C30-C31-C36	-7.5(11)	C29-C30-C31-C32	-10.2(14)
C2-C1-C5-Ru1	-73.3(11)	C29-C30-C31-C36	171.9(8)
C2-C1-C5-C6	-160.3(10)	C30-N2-C26-C27	0.0(13)
C2-C1-C5-C9	17.0(16)	C30-C31-C32-C33	-176.8(9)

C5-C1-C2-C3	172.1(10)	C30-C31-C36-Ir1	5.7(10)
C5-C6-C7-Ru1	-61.4(7)	C30-C31-C36-C35	178.4(8)
C5-C6-C7-C8	0.9(13)	C31-C32-C33-C34	-0.4(15)
C6-C5-C9-Ru1	61.3(7)	C32-C31-C36-Ir1	-172.2(7)
C6-C5-C9-C8	-1.3(12)	C32-C31-C36-C35	0.5(13)
C6-C7-C8-Ru1	-61.7(8)	C32-C33-C34-C35	-1.7(16)
C6-C7-C8-C9	-1.7(13)	C33-C34-C35-C36	3.3(16)
C7-C8-C9-Ru1	-60.8(8)	C34-C35-C36-Ir1	169.0(8)
C7-C8-C9-C5	1.9(13)	C34-C35-C36-C31	-2.6(15)
C9-C5-C6-Ru1	-61.2(7)	C36-C31-C32-C33	1.0(14)
C9-C5-C6-C7	0.3(12)	Cl1-C37-Cl2-Cl1^{1}	-8(4)
C10-C11-C12-Ru1	61.4(7)	Cl2-C37-Cl1-^{Cl21}	16(8)

^{1}2-X,-Y,1-Z

Table S7 Hydrogen Bonds (Å, º)

D-H...*A*	*D*-H	H...*A*	*D*...*A*	*D*-H...*A*
C15-H15 .. O1	0.9500	2.5000	3.0827	119.00 .
C26-H26 .. O2	0.9500	2.5600	3.1469	120.00 .

Table S8 Atomic Occupancy for **3**.

Atom	Occupancy	Atom	Occupancy	Atom	Occupancy
C37	0.333(14)	H37A	0.333(14)	H37B	0.333(14)
Cl1	0.333(14)	Cl2	0.333(14)		

SCXRD Precession images:

Shown below are calculated precession images of the complete data set (10 set of runs). Each layer has been calculated to a pixel thickness of 0.1 at a resolution of up to 0.80 Å.

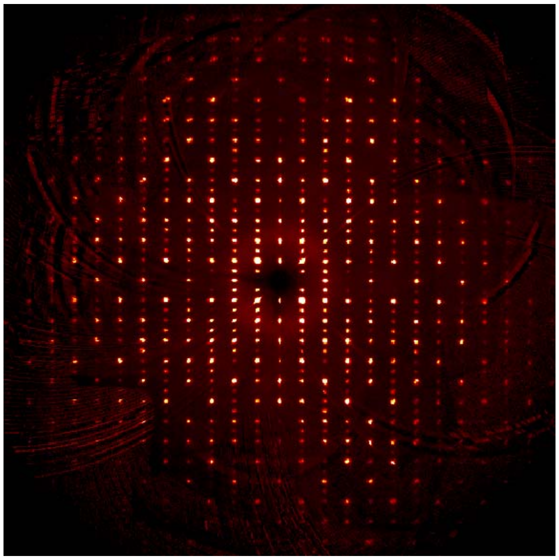

(a) Precession image of the 0kl layer plane.

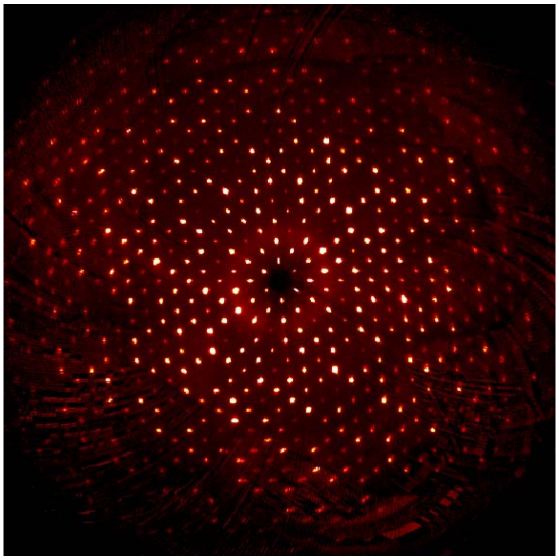

(b) Precession image of the h0l layer plane.

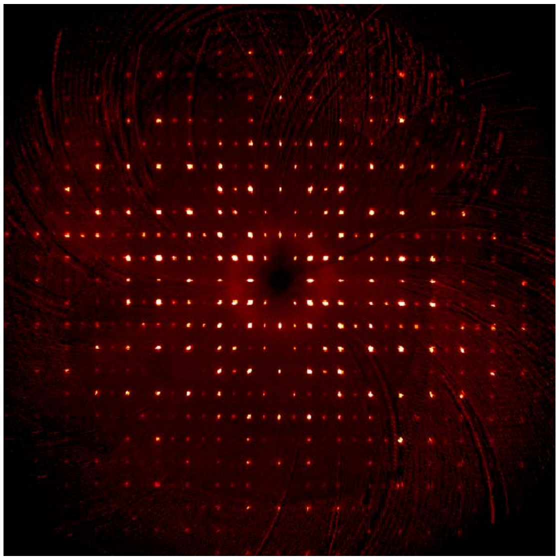

(c) Precession image of the hk0 layer plane.

F. DFT Figures.

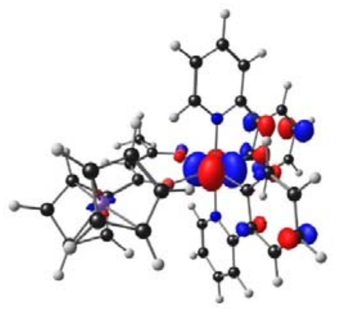	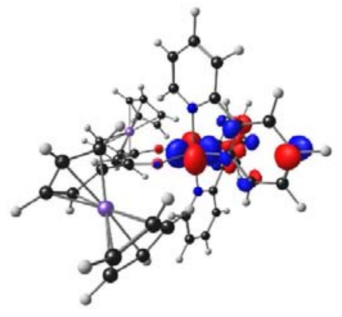	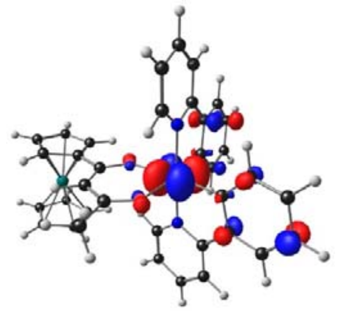
**1**$^+$, [(ppy)$_2$Ir^{III}(Fc$^+$COCHCOCH$_3$)]	**2**$^{2+}$, [(ppy)$_2$Ir^{III}(Fc$^+$COCHCOFc$^+$)]	**3**, [(ppy)$_2$Ir^{III}(RuCOCHCOCH$_3$)]
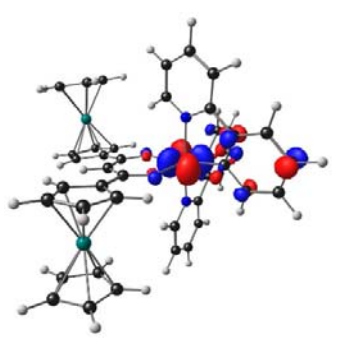	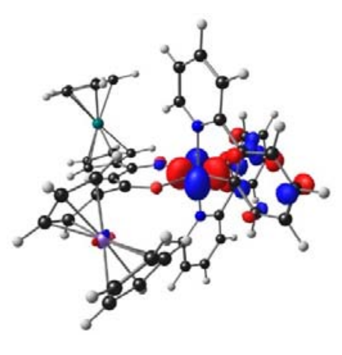	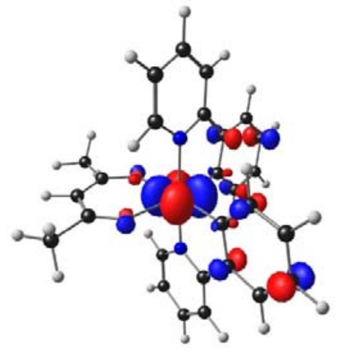
**4**, [(ppy)$_2$Ir^{III}(RuCOCHCORu)]	**5**$^+$, [(ppy)$_2$Ir^{III}(Fc$^+$COCHCORu)]	**6**, [(ppy)$_2$Ir^{III}(CH$_3$COCHCOCH$_3$)]

Fig. S2 Gas phase B3LYP/6-311G(d,p)/def2-TZVPP(SDD) calculated HOMOs of molecules involved in the Ir^{III} oxidation. HOMOs obtained from solvent DCM calculation looked similar. A contour of 0.06 eÅ^{-3} was used for the MO plots.

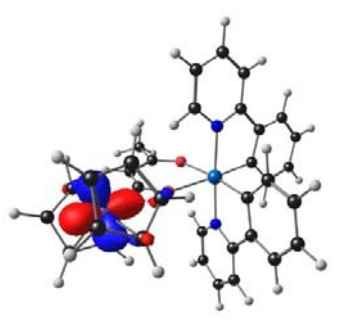	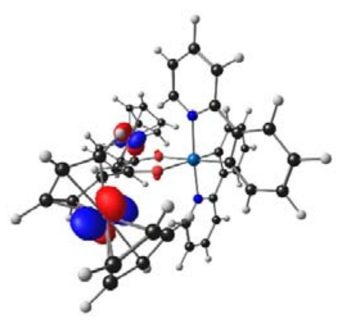	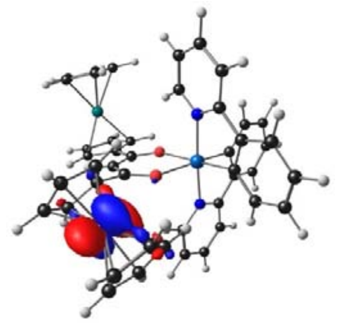
**1**$^+$, [(ppy)$_2$Ir^{III}(Fc$^+$COCHCOCH$_3$)]	**2**$^{2+}$, [(ppy)$_2$Ir^{III}(Fc$^+$COCHCOFc$^+$)]	**5**$^+$, [(ppy)$_2$Ir^{III}(Fc$^+$COCHCORu)]

Fig. S3 Gas phase B3LYP/6-311G(d,p)/def2-TZVPP(SDD) calculated LUMOs of molecules after ferrocenyl oxidation. LUMOs obtained from solvent DCM calculation looked similar. A contour of 0.06 eÅ^{-3} was used for the MO plots.

G. DFT data.

Table S9 DFT calculated HOMO energies of the indicated species

**Molecule**	$E^{o'}$/V	$E_{HOMO, gas\ phase}$ / eV	$E_{HOMO, DCM}$ / eV
1^+	0.497	-7.334	-5.602
2^{2+}	0.681	-9.458	-5.933
3	0.283	-4.992	-5.289
4	0.252	-4.989	-5.292
5^+	0.445	-7.213	-5.585
6	0.318	-5.001	-5.291

H. DFT Optimized Coordinates.

1^+, $[(ppy)_2Ir^{III}(Fc^+COCHCOCH_3)]$ gas phase

```
Ir      -1.204026000      0.036970000     -0.222070000
Fe       4.264070000     -0.251165000      0.154779000
O        0.981555000      0.017292000     -0.560315000
O       -1.319486000     -0.770862000     -2.272604000
N       -1.377826000     -1.930348000      0.401738000
N       -1.134271000      2.021603000     -0.780559000
C        1.562522000     -0.385620000     -1.618727000
C        1.013094000     -0.967021000     -2.757420000
H        1.698775000     -1.263236000     -3.540336000
C       -0.380057000     -1.130488000     -3.026752000
C       -0.780723000     -1.792651000     -4.325766000
H       -1.689451000     -1.327194000     -4.708934000
H       -1.013063000     -2.844322000     -4.127400000
H        0.006375000     -1.752756000     -5.079297000
C        3.042225000     -0.140476000     -1.619559000
C        3.641471000      1.115280000     -1.286103000
H        3.095320000      2.016147000     -1.052046000
C        5.058509000      0.960165000     -1.329610000
H        5.790190000      1.732437000     -1.145136000
C        5.336606000     -0.400634000     -1.651313000
H        6.315649000     -0.849392000     -1.731951000
C        4.094789000     -1.071374000     -1.831089000
H        3.966402000     -2.121874000     -2.047868000
C        3.103105000     -0.283484000      1.960124000
H        2.056073000     -0.016185000      1.985124000
C        4.192418000      0.608756000      2.118759000
```

H	4.113074000	1.668232000	2.312442000
C	5.400841000	-0.117816000	1.925477000
H	6.399787000	0.289864000	1.969690000
C	5.057767000	-1.472261000	1.643593000
H	5.750663000	-2.278733000	1.456917000
C	3.632562000	-1.569513000	1.660253000
H	3.055972000	-2.463678000	1.479447000
C	-0.350127000	-2.787387000	0.533989000
H	0.626856000	-2.372159000	0.325203000
C	-0.520559000	-4.108083000	0.906208000
H	0.336518000	-4.762410000	0.998304000
C	-1.817426000	-4.565228000	1.150972000
H	-1.992631000	-5.593276000	1.445043000
C	-2.879610000	-3.690206000	1.010282000
H	-3.890812000	-4.027310000	1.191446000
C	-2.655457000	-2.359291000	0.629139000
C	-3.675096000	-1.335629000	0.442207000
C	-5.046412000	-1.564583000	0.633756000
H	-5.408064000	-2.543015000	0.929663000
C	-5.955937000	-0.535784000	0.445580000
H	-7.014865000	-0.710055000	0.593358000
C	-5.493984000	0.727567000	0.068155000
H	-6.201856000	1.537311000	-0.074223000
C	-4.134565000	0.960932000	-0.123751000
H	-3.812983000	1.955245000	-0.410056000
C	-3.191165000	-0.059743000	0.051194000
C	-1.337675000	2.464688000	-2.033932000
H	-1.577101000	1.703792000	-2.764955000
C	-1.263183000	3.806453000	-2.366380000
H	-1.441164000	4.119162000	-3.386764000
C	-0.968531000	4.725728000	-1.359257000
H	-0.908756000	5.784681000	-1.581304000
C	-0.766163000	4.272113000	-0.065225000
H	-0.554303000	4.972794000	0.731021000
C	-0.854804000	2.904886000	0.222653000
C	-0.705375000	2.284546000	1.538179000
C	-0.401186000	3.002005000	2.704158000
H	-0.244201000	4.074631000	2.667843000
C	-0.307747000	2.345219000	3.923601000
H	-0.082760000	2.900721000	4.826271000
C	-0.528523000	0.966650000	3.979018000
H	-0.477477000	0.452445000	4.933246000
C	-0.829217000	0.247697000	2.822948000
H	-1.012470000	-0.817986000	2.902448000
C	-0.918618000	0.879503000	1.573472000

2^{2+}, $[(ppy)_2Ir^{III}(Fc^+COCHCOFc^+)]$ gas phase

Ir	0.000040000	1.132454000	0.000001000
Fe	4.441259000	-2.257343000	-0.118145000

Fe	-4.441450000	-2.257139000	0.118091000
O	-1.482582000	-0.525868000	-0.170358000
O	1.482505000	-0.526017000	0.170240000
N	-0.101489000	1.208795000	-2.069067000
C	-0.664762000	0.483604000	2.903898000
H	-1.382177000	-0.169543000	2.424654000
C	-0.557029000	0.567270000	4.280084000
H	-1.198233000	-0.031066000	4.913929000
C	0.386623000	1.444343000	4.819082000
H	0.493906000	1.545781000	5.892392000
C	1.181136000	2.190097000	3.965061000
H	1.911060000	2.880182000	4.365367000
C	1.035763000	2.067138000	2.576418000
C	1.795156000	2.793116000	1.563777000
C	2.814716000	3.708438000	1.866240000
H	3.087236000	3.913525000	2.895386000
C	3.481495000	4.370965000	0.845841000
H	4.261619000	5.085368000	1.078616000
C	3.125195000	4.122808000	-0.481540000
H	3.631762000	4.652174000	-1.281414000
C	2.112046000	3.214945000	-0.789337000
H	1.847334000	3.063686000	-1.829212000
C	-1.425486000	2.526481000	-0.219284000
C	-2.111739000	3.215122000	0.789479000
H	-1.847054000	3.063753000	1.829345000
C	-3.124778000	4.123128000	0.481742000
H	-3.631288000	4.652494000	1.281653000
C	-3.481039000	4.371428000	-0.845622000
H	-4.261077000	5.085941000	-1.078349000
C	-2.814333000	3.708896000	-1.866066000
H	-3.086824000	3.914090000	-2.895198000
C	-1.794886000	2.793429000	-1.563664000
C	-1.035577000	2.067432000	-2.576354000
C	-1.180939000	2.190499000	-3.964988000
H	-1.910784000	2.880696000	-4.365247000
C	-0.386518000	1.444707000	-4.819061000
H	-0.493793000	1.546228000	-5.892364000
C	0.557030000	0.567486000	-4.280123000
H	1.198159000	-0.030888000	-4.914010000
C	0.664759000	0.483717000	-2.903943000
H	1.382096000	-0.169550000	-2.424748000
C	1.237295000	-1.763137000	0.198115000
C	-0.000119000	-2.409563000	-0.000170000
H	-0.000167000	-3.491465000	-0.000224000
C	-1.237477000	-1.763005000	-0.198387000
C	2.417605000	-2.631057000	0.526037000
C	2.902282000	-3.776416000	-0.163330000
H	2.483461000	-4.190953000	-1.069031000
C	4.067435000	-4.244370000	0.503030000
H	4.670350000	-5.089059000	0.203400000
C	4.309320000	-3.391921000	1.619743000

H	5.113727000	-3.491785000	2.333186000
C	3.302595000	-2.382203000	1.623103000
H	3.202923000	-1.571266000	2.328304000
C	4.743570000	-0.275328000	-0.864584000
H	4.047789000	0.532224000	-0.685822000
C	4.675101000	-1.223648000	-1.924247000
H	3.951337000	-1.229486000	-2.724847000
C	5.736785000	-2.162789000	-1.749172000
H	5.962638000	-2.999733000	-2.392794000
C	6.452965000	-1.790628000	-0.574655000
H	7.303460000	-2.309082000	-0.156945000
C	5.838131000	-0.627139000	-0.035428000
H	6.128184000	-0.121622000	0.874070000
C	-2.417865000	-2.630789000	-0.526379000
C	-3.302943000	-2.381641000	-1.623304000
H	-3.203293000	-1.570543000	-2.328323000
C	-4.309721000	-3.391304000	-1.620083000
H	-5.114195000	-3.490960000	-2.333480000
C	-4.067782000	-4.244020000	-0.503585000
H	-4.670711000	-5.088747000	-0.204094000
C	-2.902544000	-3.776275000	0.162778000
H	-2.483665000	-4.191038000	1.068349000
C	-4.743732000	-0.275284000	0.864813000
H	-4.048063000	0.532342000	0.685959000
C	-5.838525000	-0.627056000	0.035943000
H	-6.128876000	-0.121455000	-0.873414000
C	-6.453128000	-1.790642000	0.575209000
H	-7.303695000	-2.309111000	0.157665000
C	-5.736585000	-2.162909000	1.749464000
H	-5.962199000	-2.999957000	2.393036000
C	-4.674902000	-1.223730000	1.924352000
H	-3.950921000	-1.229615000	2.724754000
N	0.101575000	1.208645000	2.069072000
C	1.425718000	2.526310000	0.219379000

3, [(ppy)$_2$Ir^{III}(RuCOCHCOCH$_3$)] gas phase

Ir	1.537283000	0.014460000	-0.164720000
Ru	-4.467664000	-0.166739000	0.137211000
O	-0.537460000	-0.664648000	-0.050802000
O	1.077267000	0.527616000	-2.235483000
N	1.091048000	1.962887000	0.326571000
N	2.067113000	-1.929992000	-0.593460000
C	-1.400085000	-0.662884000	-0.985653000
C	-1.217053000	-0.159933000	-2.290703000
H	-2.071081000	-0.200207000	-2.951360000
C	-0.045201000	0.401462000	-2.820526000
C	-0.069300000	0.913992000	-4.247838000
H	0.712288000	0.413800000	-4.826310000
H	0.169987000	1.981015000	-4.247607000

H	-1.031927000	0.762779000	-4.737252000
C	-2.709943000	-1.265143000	-0.623396000
C	-3.027145000	-1.743529000	0.693051000
H	-2.357294000	-1.695503000	1.536294000
C	-4.333649000	-2.308857000	0.662565000
H	-4.847625000	-2.763122000	1.496226000
C	-4.840808000	-2.184241000	-0.668802000
H	-5.801706000	-2.531392000	-1.017925000
C	-3.848317000	-1.535994000	-1.462160000
H	-3.930715000	-1.324634000	-2.516627000
C	-4.414576000	1.575359000	1.486057000
H	-3.725033000	1.679846000	2.309858000
C	-5.711773000	0.977164000	1.549929000
H	-6.172338000	0.554612000	2.429891000
C	-6.298974000	1.053774000	0.248572000
H	-7.280625000	0.698942000	-0.026178000
C	-5.365891000	1.700389000	-0.620220000
H	-5.518279000	1.920218000	-1.665880000
C	-4.202052000	2.021274000	0.143731000
H	-3.317743000	2.511666000	-0.233918000
C	-0.125735000	2.387178000	0.710175000
H	-0.878818000	1.614752000	0.797155000
C	-0.393829000	3.717689000	0.982761000
H	-1.387499000	4.014625000	1.292301000
C	0.639982000	4.645930000	0.849399000
H	0.465603000	5.696784000	1.050151000
C	1.896275000	4.209301000	0.464297000
H	2.712078000	4.912395000	0.364158000
C	2.122956000	2.850353000	0.207669000
C	3.396362000	2.239670000	-0.159654000
C	4.586614000	2.966191000	-0.315277000
H	4.595789000	4.044060000	-0.191872000
C	5.767175000	2.310185000	-0.628844000
H	6.687734000	2.869665000	-0.749167000
C	5.758450000	0.920537000	-0.781437000
H	6.681941000	0.400968000	-1.017770000
C	4.579840000	0.195682000	-0.631933000
H	4.608835000	-0.881236000	-0.753375000
C	3.365135000	0.828285000	-0.327351000
C	2.158903000	-2.432399000	-1.836965000
H	1.979354000	-1.724824000	-2.635474000
C	2.469401000	-3.759269000	-2.078898000
H	2.532956000	-4.120163000	-3.097118000
C	2.691894000	-4.599792000	-0.987695000
H	2.934041000	-5.645576000	-1.138412000
C	2.605551000	-4.082747000	0.294194000
H	2.781799000	-4.717493000	1.152019000
C	2.293735000	-2.731077000	0.489706000
C	2.201813000	-2.041197000	1.772966000
C	2.410977000	-2.673792000	3.007684000
H	2.633830000	-3.734593000	3.053836000

C	2.335709000	-1.945604000	4.185426000
H	2.497723000	-2.432566000	5.140208000
C	2.055298000	-0.577362000	4.127302000
H	2.003374000	-0.000687000	5.045723000
C	1.844460000	0.054264000	2.905015000
H	1.631820000	1.117486000	2.896537000
C	1.902523000	-0.653613000	1.694614000

4, [$(ppy)_2Ir^{III}$(RuCOCHCORu)] gas phase

Ir	1.914033000	0.055690000	0.037079000
O	0.414284000	1.346119000	-0.875202000
O	0.551330000	-1.576754000	-0.433480000
N	1.056332000	0.441262000	1.868678000
N	2.870864000	-0.331825000	-1.745857000
C	0.089918000	-0.309855000	2.426197000
H	-0.209446000	-1.180015000	1.856202000
C	-0.479521000	0.003835000	3.648709000
H	-1.252076000	-0.631701000	4.061837000
C	-0.030676000	1.142435000	4.320510000
H	-0.455428000	1.420163000	5.278252000
C	0.968916000	1.913308000	3.751132000
H	1.335337000	2.794496000	4.259902000
C	1.520577000	1.552742000	2.513831000
C	2.598901000	2.243336000	1.814143000
C	3.218290000	3.403889000	2.303067000
H	2.894116000	3.852177000	3.236452000
C	4.255609000	3.991105000	1.595051000
H	4.736013000	4.886860000	1.971541000
C	4.678223000	3.412325000	0.394608000
H	5.494734000	3.863832000	-0.160470000
C	4.066075000	2.262577000	-0.095568000
H	4.421190000	1.838867000	-1.028310000
C	3.007438000	1.646440000	0.589767000
C	2.553609000	0.249122000	-2.916194000
H	1.769819000	0.993357000	-2.866667000
C	3.189611000	-0.077750000	-4.101280000
H	2.900997000	0.415661000	-5.020116000
C	4.193108000	-1.046644000	-4.072391000
H	4.711055000	-1.330346000	-4.981385000
C	4.525134000	-1.642128000	-2.866826000
H	5.304272000	-2.391040000	-2.824088000
C	3.856387000	-1.275988000	-1.691713000
C	4.116357000	-1.793245000	-0.351160000
C	5.095873000	-2.757823000	-0.071235000
H	5.697922000	-3.180863000	-0.868520000
C	5.305517000	-3.180533000	1.232829000
H	6.062591000	-3.925038000	1.450816000
C	4.534995000	-2.632254000	2.262065000
H	4.700393000	-2.953356000	3.285907000

C	3.560017000	-1.676981000	1.989106000
H	2.983454000	-1.269243000	2.811980000
C	3.314000000	-1.234151000	0.680650000
C	-0.727087000	1.000589000	-1.326431000
C	-1.228674000	-0.310490000	-1.418352000
H	-2.204922000	-0.425337000	-1.863909000
C	-0.584624000	-1.491299000	-1.004795000
C	-1.555768000	2.118599000	-1.849505000
C	-2.920011000	2.101930000	-2.308607000
H	-3.578996000	1.248155000	-2.321416000
C	-3.251202000	3.409119000	-2.773903000
H	-4.199489000	3.709935000	-3.193126000
C	-2.104346000	4.245810000	-2.598787000
H	-2.036058000	5.290240000	-2.863468000
C	-1.064566000	3.458091000	-2.024908000
H	-0.066795000	3.779135000	-1.772681000
C	-4.038941000	2.991879000	1.094526000
H	-4.687298000	2.129955000	1.135047000
C	-4.398066000	4.291607000	0.619334000
H	-5.365149000	4.583475000	0.238817000
C	-3.257651000	5.143020000	0.758833000
H	-3.211835000	6.189479000	0.498138000
C	-2.194088000	4.369638000	1.320443000
H	-1.201111000	4.724913000	1.549181000
C	-2.677276000	3.040689000	1.526733000
H	-2.108149000	2.217818000	1.931622000
C	-1.253919000	-2.797706000	-1.243632000
C	-0.735620000	-4.052018000	-0.774575000
H	0.168829000	-4.159964000	-0.198129000
C	-1.593573000	-5.092432000	-1.231843000
H	-1.462687000	-6.148557000	-1.050545000
C	-2.651119000	-4.495776000	-1.987536000
H	-3.454490000	-5.021397000	-2.481443000
C	-2.450093000	-3.083525000	-1.991619000
H	-3.070926000	-2.365860000	-2.504425000
C	-3.554898000	-4.772745000	1.854471000
H	-3.376645000	-5.812234000	2.084020000
C	-4.642584000	-4.256962000	1.082931000
H	-5.430105000	-4.838934000	0.628812000
C	-4.515621000	-2.833861000	1.036046000
H	-5.188568000	-2.152189000	0.538704000
C	-3.349470000	-2.470777000	1.777407000
H	-2.982997000	-1.465771000	1.921883000
C	-2.754538000	-3.668647000	2.284140000
H	-1.866937000	-3.731040000	2.895377000
Ru	-2.747697000	-3.743056000	0.081440000
Ru	-2.756262000	3.506789000	-0.625422000

5^+, $[(ppy)_2Ir^{III}(Fc^+COCHCORu)]$ gas phase

Ir	-0.736242000	-1.328218000	0.069580000
Ru	4.815325000	1.021022000	-0.142789000
Fe	-3.077701000	3.633206000	-0.270653000
O	-1.242056000	0.818933000	0.083836000
O	1.259571000	-0.650369000	-0.561867000
N	-0.393259000	-1.128155000	2.091787000
C	-1.542188000	-0.711016000	-2.799174000
H	-1.746551000	0.255414000	-2.357937000
C	-1.721286000	-0.955747000	-4.148274000
H	-2.077484000	-0.169729000	-4.801361000
C	-1.425299000	-2.230549000	-4.635347000
H	-1.552263000	-2.461773000	-5.686293000
C	-0.962704000	-3.197157000	-3.760240000
H	-0.723906000	-4.188365000	-4.120147000
C	-0.796291000	-2.899696000	-2.400158000
C	-0.317777000	-3.814931000	-1.371819000
C	0.037587000	-5.148411000	-1.626841000
H	-0.025766000	-5.555784000	-2.629774000
C	0.474131000	-5.961811000	-0.592769000
H	0.747558000	-6.991921000	-0.787176000
C	0.552305000	-5.442330000	0.701865000
H	0.886278000	-6.077472000	1.515688000
C	0.201605000	-4.119728000	0.960813000
H	0.268107000	-3.753881000	1.978701000
C	-2.564754000	-1.764596000	0.762845000
C	-3.707673000	-2.122659000	0.031747000
H	-3.635091000	-2.279248000	-1.038728000
C	-4.940706000	-2.304487000	0.656154000
H	-5.804491000	-2.592352000	0.065638000
C	-5.072457000	-2.135090000	2.036950000
H	-6.031367000	-2.285866000	2.518432000
C	-3.958365000	-1.792505000	2.790634000
H	-4.061618000	-1.675859000	3.864104000
C	-2.714833000	-1.609787000	2.168262000
C	-1.489037000	-1.279310000	2.892562000
C	-1.355443000	-1.140839000	4.279737000
H	-2.221475000	-1.269504000	4.914788000
C	-0.117402000	-0.860111000	4.835420000
H	-0.011364000	-0.761946000	5.909390000
C	0.990146000	-0.719293000	3.998311000
H	1.976640000	-0.513950000	4.392808000
C	0.808767000	-0.861202000	2.633022000
H	1.627625000	-0.775466000	1.930318000
C	1.653443000	0.517559000	-0.836189000
C	0.868857000	1.707674000	-0.672436000
H	1.362038000	2.649595000	-0.866688000
C	-0.434766000	1.766972000	-0.203741000
C	3.022880000	0.640590000	-1.365007000
C	3.702977000	1.814377000	-1.859178000
H	3.301273000	2.813165000	-1.926513000
C	4.994006000	1.416409000	-2.309475000

H	5.736224000	2.064630000	-2.749912000
C	5.132253000	0.010679000	-2.087173000
H	6.000600000	-0.583857000	-2.326759000
C	3.927589000	-0.469649000	-1.504730000
H	3.697193000	-1.487537000	-1.235020000
C	4.444399000	1.383592000	2.004912000
H	3.461166000	1.423191000	2.449242000
C	5.159855000	2.496230000	1.465423000
H	4.819733000	3.520158000	1.441169000
C	6.419010000	2.018410000	0.988614000
H	7.194763000	2.617054000	0.536278000
C	6.480574000	0.611605000	1.234332000
H	7.310315000	-0.037161000	0.999083000
C	5.259238000	0.217676000	1.863141000
H	5.008927000	-0.780810000	2.187473000
C	-1.002580000	3.127764000	0.060945000
C	-1.682815000	3.485184000	1.267893000
H	-1.808240000	2.833560000	2.118876000
C	-2.144666000	4.828603000	1.142288000
H	-2.675591000	5.393040000	1.894188000
C	-1.788765000	5.289806000	-0.159236000
H	-2.020247000	6.258007000	-0.578047000
C	-1.087702000	4.241211000	-0.818710000
H	-0.717942000	4.264895000	-1.833444000
C	-4.069084000	2.576948000	-1.784505000
H	-3.578038000	2.161222000	-2.650862000
C	-4.306257000	1.900577000	-0.554974000
H	-3.970862000	0.905367000	-0.299550000
C	-4.968582000	2.801536000	0.315603000
H	-5.246959000	2.595559000	1.338536000
C	-5.150739000	4.035828000	-0.367922000
H	-5.612017000	4.923504000	0.038678000
C	-4.595878000	3.900515000	-1.673485000
H	-4.581901000	4.659694000	-2.440789000
N	-1.099764000	-1.648258000	-1.942870000
C	-0.236184000	-3.269632000	-0.063504000

6, [(ppy)$_2$Ir^{III}(CH$_3$COCHCOCH$_3$)] gas phase

Ir	0.000006000	0.149647000	-0.000023000
O	-0.642643000	1.756154000	1.338176000
O	0.642040000	1.756439000	-1.338144000
N	1.934574000	0.098152000	0.706705000
N	-1.934606000	0.097715000	-0.706703000
C	-0.574492000	3.004650000	1.119893000
C	-0.000683000	3.634468000	0.000034000
H	-0.000880000	4.716169000	0.000052000
C	0.573418000	3.004909000	-1.119818000
C	1.175642000	3.871749000	-2.208583000
H	0.718180000	3.619354000	-3.168937000

H	2.241844000	3.644193000	-2.295237000
H	1.052139000	4.938112000	-2.018008000
C	-1.176865000	3.871229000	2.208796000
C	2.377396000	0.816533000	1.753030000
H	1.627151000	1.411994000	2.255918000
C	3.698602000	0.790452000	2.164441000
H	4.009791000	1.384672000	3.013657000
C	4.598236000	-0.011252000	1.460796000
H	5.641549000	-0.054443000	1.751776000
C	4.143098000	-0.758138000	0.387045000
H	4.823927000	-1.390733000	-0.166200000
C	2.794317000	-0.704483000	0.011538000
C	2.166364000	-1.457874000	-1.069102000
C	2.862671000	-2.354599000	-1.893453000
H	3.929614000	-2.506264000	-1.767770000
C	2.190187000	-3.059862000	-2.879818000
H	2.726088000	-3.753979000	-3.516947000
C	0.814311000	-2.870730000	-3.039242000
H	0.281432000	-3.426476000	-3.804642000
C	0.119713000	-1.979944000	-2.226165000
H	-0.947642000	-1.860248000	-2.374249000
C	0.769967000	-1.242929000	-1.224915000
C	-2.377681000	0.816006000	-1.752974000
H	-1.627630000	1.411694000	-2.255880000
C	-3.698906000	0.789586000	-2.164310000
H	-4.010298000	1.383739000	-3.013499000
C	-4.598282000	-0.012369000	-1.460630000
H	-5.641602000	-0.055831000	-1.751545000
C	-4.142883000	-0.759161000	-0.386920000
H	-4.823517000	-1.391951000	0.166339000
C	-2.794093000	-0.705164000	-0.011500000
C	-2.165888000	-1.458457000	1.069070000
C	-2.861948000	-2.355363000	1.893431000
H	-3.928873000	-2.507227000	1.767840000
C	-2.189245000	-3.060552000	2.879702000
H	-2.724963000	-3.754802000	3.516840000
C	-0.813394000	-2.871168000	3.039017000
H	-0.280338000	-3.426865000	3.804329000
C	-0.119040000	-1.980191000	2.225940000
H	0.948303000	-1.860297000	2.373944000
C	-0.769522000	-1.243228000	1.224802000
H	-2.242624000	3.642150000	2.296698000
H	-0.718019000	3.620039000	3.168815000
H	-1.055051000	4.937661000	2.017536000

1^+, $[(ppy)_2Ir^{III}(Fc^+COCHCOCH_3)]$ DCM

Ir	-1.286172000	0.018156000	-0.189918000
Fe	4.581255000	-0.045789000	0.140739000
O	0.892153000	0.361918000	-0.258724000

O	-1.091320000	-0.491306000	-2.316424000
N	-1.183209000	-2.013909000	0.174885000
N	-1.484957000	2.054053000	-0.485997000
C	1.620005000	0.294569000	-1.299361000
C	1.258630000	-0.132955000	-2.581858000
H	2.032134000	-0.134812000	-3.336482000
C	-0.038071000	-0.508229000	-3.015777000
C	-0.215715000	-0.946859000	-4.452453000
H	-0.892042000	-0.252654000	-4.959369000
H	-0.692235000	-1.930233000	-4.470363000
H	0.724647000	-0.991645000	-5.000766000
C	3.019666000	0.793263000	-1.116604000
C	3.405897000	1.701964000	-0.087365000
H	2.744800000	2.082205000	0.675326000
C	4.790994000	1.989467000	-0.234535000
H	5.372600000	2.645391000	0.394918000
C	5.277430000	1.236303000	-1.345791000
H	6.294819000	1.212644000	-1.705412000
C	4.187234000	0.493200000	-1.878874000
H	4.245872000	-0.206553000	-2.698338000
C	3.917682000	-1.321857000	1.654670000
H	2.907761000	-1.307215000	2.034242000
C	5.006764000	-0.555068000	2.159028000
H	4.959976000	0.155125000	2.970557000
C	6.152278000	-0.844864000	1.372336000
H	7.122642000	-0.382501000	1.473529000
C	5.779499000	-1.784725000	0.376436000
H	6.422387000	-2.171574000	-0.399612000
C	4.397777000	-2.086036000	0.547521000
H	3.819352000	-2.760963000	-0.064282000
C	-0.039206000	-2.696122000	0.357373000
H	0.862789000	-2.101203000	0.337074000
C	-0.013092000	-4.064836000	0.560802000
H	0.932044000	-4.570851000	0.705718000
C	-1.224142000	-4.757598000	0.572759000
H	-1.244295000	-5.829450000	0.728519000
C	-2.405613000	-4.058466000	0.383647000
H	-3.352869000	-4.579786000	0.390416000
C	-2.379039000	-2.672973000	0.184165000
C	-3.539479000	-1.806448000	-0.016551000
C	-4.861468000	-2.276151000	-0.036229000
H	-5.074520000	-3.331751000	0.091396000
C	-5.913794000	-1.389212000	-0.219616000
H	-6.935280000	-1.751074000	-0.234337000
C	-5.642823000	-0.027589000	-0.381277000
H	-6.461828000	0.670885000	-0.520604000
C	-4.331095000	0.443457000	-0.363011000
H	-4.157339000	1.506222000	-0.487951000
C	-3.244334000	-0.426613000	-0.186023000
C	-1.582200000	2.641300000	-1.692264000
H	-1.586351000	1.971938000	-2.541702000

C	-1.682082000	4.013851000	-1.842868000
H	-1.760829000	4.441510000	-2.833416000
C	-1.681370000	4.810795000	-0.698192000
H	-1.758683000	5.888491000	-0.777235000
C	-1.587433000	4.208162000	0.546747000
H	-1.594080000	4.811032000	1.444470000
C	-1.491496000	2.815604000	0.647388000
C	-1.413852000	2.041865000	1.887393000
C	-1.405685000	2.624259000	3.163770000
H	-1.441733000	3.701896000	3.279505000
C	-1.353417000	1.822147000	4.296443000
H	-1.349171000	2.271723000	5.282612000
C	-1.313932000	0.432531000	4.152102000
H	-1.281216000	-0.199258000	5.034117000
C	-1.320113000	-0.150474000	2.885684000
H	-1.293474000	-1.231774000	2.808688000
C	-1.363194000	0.631145000	1.720757000

2^{2+}, $[(ppy)_2Ir^{III}(Fc^{+}COCHCOFc^{+})]$ DCM

Ir	0.000000000	0.000000000	0.000000000
Fe	4.441219000	-3.389797000	-0.118146000
Fe	-4.441490000	-3.389593000	0.118090000
O	-1.482622000	-1.658322000	-0.170359000
O	1.482465000	-1.658471000	0.170239000
N	-0.101529000	0.076341000	-2.069068000
C	-0.664802000	-0.648850000	2.903897000
H	-1.382217000	-1.301997000	2.424653000
C	-0.557069000	-0.565184000	4.280083000
H	-1.198273000	-1.163520000	4.913928000
C	0.386583000	0.311889000	4.819081000
H	0.493866000	0.413327000	5.892391000
C	1.181096000	1.057643000	3.965060000
H	1.911020000	1.747728000	4.365366000
C	1.035723000	0.934684000	2.576417000
C	1.795116000	1.660662000	1.563776000
C	2.814676000	2.575984000	1.866239000
H	3.087196000	2.781071000	2.895385000
C	3.481455000	3.238511000	0.845840000
H	4.261579000	3.952914000	1.078615000
C	3.125155000	2.990354000	-0.481541000
H	3.631722000	3.519720000	-1.281415000
C	2.112006000	2.082491000	-0.789338000
H	1.847294000	1.931232000	-1.829213000
C	-1.425526000	1.394027000	-0.219285000
C	-2.111779000	2.082668000	0.789478000
H	-1.847094000	1.931299000	1.829344000
C	-3.124818000	2.990674000	0.481741000
H	-3.631328000	3.520040000	1.281652000
C	-3.481079000	3.238974000	-0.845623000

H	-4.261117000	3.953487000	-1.078350000
C	-2.814373000	2.576442000	-1.866067000
H	-3.086864000	2.781636000	-2.895199000
C	-1.794926000	1.660975000	-1.563665000
C	-1.035617000	0.934978000	-2.576355000
C	-1.180979000	1.058045000	-3.964989000
H	-1.910824000	1.748242000	-4.365248000
C	-0.386558000	0.312253000	-4.819062000
H	-0.493833000	0.413774000	-5.892365000
C	0.556990000	-0.564968000	-4.280124000
H	1.198119000	-1.163342000	-4.914011000
C	0.664719000	-0.648737000	-2.903944000
H	1.382056000	-1.302004000	-2.424749000
C	1.237255000	-2.895591000	0.198114000
C	-0.000159000	-3.542017000	-0.000171000
H	-0.000207000	-4.623919000	-0.000225000
C	-1.237517000	-2.895459000	-0.198388000
C	2.417565000	-3.763511000	0.526036000
C	2.902242000	-4.908870000	-0.163331000
H	2.483421000	-5.323407000	-1.069032000
C	4.067395000	-5.376824000	0.503029000
H	4.670310000	-6.221513000	0.203399000
C	4.309280000	-4.524375000	1.619742000
H	5.113687000	-4.624239000	2.333185000
C	3.302555000	-3.514657000	1.623102000
H	3.202883000	-2.703720000	2.328303000
C	4.743530000	-1.407782000	-0.864585000
H	4.047749000	-0.600230000	-0.685823000
C	4.675061000	-2.356102000	-1.924248000
H	3.951297000	-2.361940000	-2.724848000
C	5.736745000	-3.295243000	-1.749173000
H	5.962598000	-4.132187000	-2.392795000
C	6.452925000	-2.923082000	-0.574656000
H	7.303420000	-3.441536000	-0.156946000
C	5.838091000	-1.759593000	-0.035429000
H	6.128144000	-1.254076000	0.874069000
C	-2.417905000	-3.763243000	-0.526380000
C	-3.302983000	-3.514095000	-1.623305000
H	-3.203333000	-2.702997000	-2.328324000
C	-4.309761000	-4.523758000	-1.620084000
H	-5.114235000	-4.623414000	-2.333481000
C	-4.067822000	-5.376474000	-0.503586000
H	-4.670751000	-6.221201000	-0.204095000
C	-2.902584000	-4.908729000	0.162777000
H	-2.483705000	-5.323492000	1.068348000
C	-4.743772000	-1.407738000	0.864812000
H	-4.048103000	-0.600112000	0.685958000
C	-5.838565000	-1.759510000	0.035942000
H	-6.128916000	-1.253909000	-0.873415000
C	-6.453168000	-2.923096000	0.575208000
H	-7.303735000	-3.441565000	0.157664000

C	-5.736625000	-3.295363000	1.749463000
H	-5.962239000	-4.132411000	2.393035000
C	-4.674942000	-2.356184000	1.924351000
H	-3.950961000	-2.362069000	2.724753000
N	0.101535000	0.076191000	2.069071000
C	1.425678000	1.393856000	0.219378000

3, [$(ppy)_2Ir^{III}(RuCOCHCOCH_3)$] DCM

Ir	1.540555000	0.016226000	-0.170505000
Ru	-4.503711000	-0.145513000	0.149605000
O	-0.546074000	-0.624882000	-0.013211000
O	1.061053000	0.528377000	-2.238555000
N	1.156195000	1.985112000	0.309208000
N	2.012934000	-1.951786000	-0.582576000
C	-1.414148000	-0.639627000	-0.945825000
C	-1.233681000	-0.163130000	-2.261589000
H	-2.089630000	-0.218436000	-2.918595000
C	-0.068030000	0.390368000	-2.813463000
C	-0.109207000	0.875010000	-4.248613000
H	0.660895000	0.358851000	-4.828671000
H	0.130854000	1.941802000	-4.274702000
H	-1.078989000	0.717089000	-4.720296000
C	-2.723685000	-1.236222000	-0.574252000
C	-3.050827000	-1.694285000	0.748649000
H	-2.397238000	-1.626278000	1.603121000
C	-4.349886000	-2.277509000	0.714312000
H	-4.867171000	-2.723493000	1.550417000
C	-4.842906000	-2.184442000	-0.625028000
H	-5.794865000	-2.551054000	-0.978734000
C	-3.850581000	-1.538342000	-1.419940000
H	-3.924783000	-1.352322000	-2.479865000
C	-4.480082000	1.635668000	1.452256000
H	-3.783090000	1.787206000	2.262440000
C	-5.758557000	1.002439000	1.551139000
H	-6.195315000	0.591563000	2.448832000
C	-6.366528000	1.028124000	0.256702000
H	-7.342557000	0.640458000	0.006661000
C	-5.464713000	1.678448000	-0.642587000
H	-5.639609000	1.868074000	-1.690785000
C	-4.300036000	2.052797000	0.096041000
H	-3.438987000	2.567469000	-0.302588000
C	-0.048191000	2.452925000	0.683093000
H	-0.832715000	1.712019000	0.760483000
C	-0.269977000	3.791653000	0.958416000
H	-1.255377000	4.122926000	1.258116000
C	0.797453000	4.682215000	0.840292000
H	0.660246000	5.737120000	1.045649000
C	2.040913000	4.201606000	0.460827000
H	2.880821000	4.876761000	0.369841000

C	2.216807000	2.837975000	0.197566000
C	3.469382000	2.185388000	-0.183868000
C	4.680336000	2.874413000	-0.349366000
H	4.727354000	3.949112000	-0.210836000
C	5.834803000	2.183857000	-0.693978000
H	6.770619000	2.715583000	-0.822159000
C	5.777691000	0.798113000	-0.870160000
H	6.677891000	0.252187000	-1.134980000
C	4.576414000	0.110233000	-0.708468000
H	4.567568000	-0.964724000	-0.851491000
C	3.388613000	0.777352000	-0.368012000
C	2.049031000	-2.481222000	-1.818534000
H	1.848292000	-1.792239000	-2.627537000
C	2.331799000	-3.817389000	-2.045250000
H	2.350409000	-4.198488000	-3.057628000
C	2.586426000	-4.639697000	-0.947683000
H	2.809484000	-5.690825000	-1.086420000
C	2.554946000	-4.096054000	0.327047000
H	2.754785000	-4.718234000	1.188724000
C	2.267559000	-2.737735000	0.504962000
C	2.222772000	-2.024212000	1.781766000
C	2.459905000	-2.640478000	3.019845000
H	2.676179000	-3.701777000	3.076138000
C	2.419269000	-1.894132000	4.190277000
H	2.602548000	-2.369763000	5.146904000
C	2.143824000	-0.525282000	4.121158000
H	2.115616000	0.064333000	5.032273000
C	1.905916000	0.090106000	2.893490000
H	1.696505000	1.154067000	2.875680000
C	1.931695000	-0.635109000	1.691096000

4, [(ppy)$_2$Ir^{III}(RuCOCHCORu)] DCM

Ir	-0.035349000	1.877305000	-0.000278000
Ru	3.930360000	-2.694496000	0.248853000
Ru	-3.822664000	-2.836583000	-0.246886000
O	-1.318607000	0.249641000	-0.689119000
O	1.315475000	0.304647000	0.689705000
N	0.752011000	1.951795000	-1.906344000
C	-1.871043000	1.180149000	2.310928000
H	-2.320556000	0.554708000	1.551261000
C	-2.348087000	1.219206000	3.610230000
H	-3.195548000	0.607827000	3.890639000
C	-1.714081000	2.056776000	4.528266000
H	-2.058627000	2.111032000	5.554017000
C	-0.638262000	2.825375000	4.112432000
H	-0.139102000	3.484894000	4.809204000
C	-0.196163000	2.755978000	2.786008000
C	0.899833000	3.527015000	2.198752000
C	1.674148000	4.444758000	2.924509000

H	1.494302000	4.603875000	3.982154000
C	2.682404000	5.160778000	2.292695000
H	3.280863000	5.870023000	2.852831000
C	2.914123000	4.960046000	0.928686000
H	3.697220000	5.520971000	0.428011000
C	2.149115000	4.047198000	0.204925000
H	2.355112000	3.916635000	-0.851759000
C	-1.259763000	3.245298000	-0.817167000
C	-2.316848000	3.944452000	-0.212322000
H	-2.519920000	3.804561000	0.843731000
C	-3.120682000	4.821296000	-0.938564000
H	-3.930526000	5.345351000	-0.440502000
C	-2.893617000	5.033007000	-2.301673000
H	-3.521921000	5.714389000	-2.863711000
C	-1.851664000	4.363898000	-2.930225000
H	-1.676008000	4.531227000	-3.987315000
C	-1.038802000	3.481959000	-2.202166000
C	0.090395000	2.758592000	-2.787312000
C	0.530501000	2.845533000	-4.113356000
H	0.004997000	3.483217000	-4.811053000
C	1.637451000	2.121636000	-4.527826000
H	1.980269000	2.189360000	-5.553356000
C	2.304451000	1.311218000	-3.608848000
H	3.176281000	0.734620000	-3.888145000
C	1.828611000	1.253637000	-2.309808000
H	2.303197000	0.647760000	-1.549567000
C	1.125852000	-0.955372000	0.613093000
C	0.034994000	-1.603101000	0.004317000
H	0.056830000	-2.682349000	0.005912000
C	-1.079833000	-1.001619000	-0.608130000
C	2.182605000	-1.784008000	1.251768000
C	2.193125000	-3.203862000	1.496184000
H	1.421629000	-3.906990000	1.224092000
C	3.380552000	-3.522486000	2.219450000
H	3.663020000	-4.504508000	2.568191000
C	4.118890000	-2.313836000	2.417886000
H	5.060254000	-2.224504000	2.938921000
C	3.386884000	-1.246293000	1.822872000
H	3.657908000	-0.203053000	1.817356000
C	3.927607000	-2.475430000	-1.949317000
H	3.122126000	-2.022307000	-2.507180000
C	4.040970000	-3.862044000	-1.623022000
H	3.343102000	-4.637979000	-1.899020000
C	5.253397000	-4.043828000	-0.886780000
H	5.631773000	-4.981615000	-0.508844000
C	5.888922000	-2.768795000	-0.759466000
H	6.830456000	-2.575781000	-0.267882000
C	5.069004000	-1.798281000	-1.415955000
H	5.283776000	-0.744398000	-1.507644000
C	-2.101963000	-1.872820000	-1.246585000
C	-3.320969000	-1.382441000	-1.829218000

H	-3.628541000	-0.349313000	-1.834258000
C	-4.011037000	-2.479820000	-2.419990000
H	-4.951685000	-2.427857000	-2.947351000
C	-3.231796000	-3.660070000	-2.207455000
H	-3.477318000	-4.654166000	-2.549894000
C	-2.060845000	-3.294020000	-1.479779000
H	-1.267085000	-3.967543000	-1.197095000
C	-3.838812000	-2.604786000	1.949850000
H	-3.050938000	-2.123395000	2.509122000
C	-4.998232000	-1.967291000	1.406480000
H	-5.247082000	-0.920307000	1.490528000
C	-5.783350000	-2.967262000	0.751621000
H	-6.727823000	-2.807135000	0.253909000
C	-5.108401000	-4.220678000	0.890026000
H	-5.454796000	-5.172215000	0.515806000
C	-3.906387000	-3.996184000	1.631487000
H	-3.185834000	-4.747961000	1.915941000
N	-0.824295000	1.921599000	1.906185000
C	1.127556000	3.299826000	0.813207000

5⁺, [(ppy)$_2$Ir^{III}(Fc^{+}COCHCORu)] DCM

Ir	0.825757000	-1.472734000	-0.032159000
Ru	-4.709856000	0.983762000	0.221719000
Fe	2.715603000	4.127244000	-0.050807000
O	1.337003000	0.631900000	-0.421308000
O	-1.103828000	-0.685229000	0.657870000
N	0.295056000	-1.594017000	-2.024791000
C	1.975935000	-0.417389000	2.572265000
H	2.156444000	0.447445000	1.949149000
C	2.316604000	-0.443422000	3.913490000
H	2.778986000	0.421773000	4.369639000
C	2.049817000	-1.601553000	4.644489000
H	2.301919000	-1.659354000	5.696468000
C	1.458733000	-2.681962000	4.009075000
H	1.247533000	-3.588070000	4.560009000
C	1.134776000	-2.607030000	2.649187000
C	0.525705000	-3.666995000	1.846599000
C	0.170215000	-4.921572000	2.364651000
H	0.327254000	-5.150838000	3.412919000
C	-0.389389000	-5.884905000	1.535959000
H	-0.663937000	-6.854372000	1.935186000
C	-0.591296000	-5.593225000	0.184000000
H	-1.023703000	-6.344268000	-0.469501000
C	-0.241453000	-4.347535000	-0.334486000
H	-0.409661000	-4.155855000	-1.388267000
C	2.570629000	-2.091259000	-0.807701000
C	3.768361000	-2.378284000	-0.134341000
H	3.804341000	-2.325704000	0.948133000
C	4.923927000	-2.735854000	-0.827284000

H	5.834232000	-2.952957000	-0.277381000
C	4.920432000	-2.821151000	-2.222421000
H	5.820836000	-3.099901000	-2.757427000
C	3.749256000	-2.552552000	-2.918820000
H	3.747945000	-2.624987000	-4.000839000
C	2.583215000	-2.194572000	-2.225497000
C	1.304542000	-1.923418000	-2.883307000
C	1.050767000	-1.996025000	-4.257847000
H	1.850728000	-2.256961000	-4.937128000
C	-0.222221000	-1.742686000	-4.744211000
H	-0.421161000	-1.801762000	-5.807501000
C	-1.241746000	-1.418863000	-3.848877000
H	-2.251107000	-1.222715000	-4.184921000
C	-0.940817000	-1.354364000	-2.498933000
H	-1.690217000	-1.114799000	-1.756483000
C	-1.480958000	0.523316000	0.717196000
C	-0.735236000	1.645611000	0.258995000
H	-1.236228000	2.601171000	0.294896000
C	0.549855000	1.625747000	-0.283292000
C	-2.811120000	0.754124000	1.322089000
C	-3.431274000	2.004150000	1.688134000
H	-3.011187000	2.990024000	1.566140000
C	-4.681109000	1.712189000	2.307602000
H	-5.371603000	2.438878000	2.708320000
C	-4.853878000	0.292519000	2.318908000
H	-5.701300000	-0.238530000	2.725279000
C	-3.709883000	-0.298907000	1.712866000
H	-3.518973000	-1.352325000	1.586834000
C	-4.512756000	1.048714000	-1.979494000
H	-3.567822000	1.032550000	-2.500964000
C	-5.205285000	2.219577000	-1.542335000
H	-4.881401000	3.239399000	-1.684637000
C	-6.415661000	1.802745000	-0.905585000
H	-7.165144000	2.452476000	-0.479694000
C	-6.470143000	0.374209000	-0.951156000
H	-7.267514000	-0.242358000	-0.564807000
C	-5.292827000	-0.092886000	-1.614780000
H	-5.047145000	-1.123754000	-1.819498000
C	1.082408000	2.913901000	-0.828497000
C	2.162385000	2.987600000	-1.756448000
H	2.719389000	2.136826000	-2.115657000
C	2.380066000	4.351955000	-2.092569000
H	3.130099000	4.725386000	-2.772889000
C	1.446074000	5.137935000	-1.352219000
H	1.364326000	6.214077000	-1.365641000
C	0.660154000	4.251468000	-0.563492000
H	-0.104996000	4.549629000	0.136457000
C	3.106998000	3.885537000	1.994385000
H	2.389199000	3.472873000	2.686599000
C	4.051512000	3.148723000	1.217202000
H	4.184875000	2.078225000	1.216778000

C	4.778833000	4.079635000	0.419536000
H	5.543282000	3.835783000	-0.302332000
C	4.289458000	5.381797000	0.706595000
H	4.603816000	6.296171000	0.226186000
C	3.258032000	5.263804000	1.673606000
H	2.661019000	6.074842000	2.062359000
N	1.402254000	-1.463720000	1.952427000
C	0.319204000	-3.348947000	0.476746000

6, [(ppy)$_2$Ir^{III}(CH$_3$COCHCOCH$_3$)] DCM

Ir	0.000000000	0.155455000	-0.000001000
O	-0.638272000	1.755971000	1.346263000
O	0.638288000	1.755958000	-1.346275000
N	1.944929000	0.103026000	0.690814000
N	-1.944929000	0.103042000	-0.690817000
C	-0.562511000	3.006958000	1.128432000
C	0.000020000	3.631539000	-0.000010000
H	0.000027000	4.713161000	-0.000012000
C	0.562544000	3.006946000	-1.128449000
C	1.139287000	3.879461000	-2.224368000
H	0.658787000	3.633288000	-3.175236000
H	2.204564000	3.657849000	-2.336484000
H	1.016642000	4.943755000	-2.024623000
C	-1.139227000	3.879485000	2.224357000
C	2.403865000	0.833263000	1.722898000
H	1.668104000	1.446540000	2.225008000
C	3.728278000	0.799120000	2.124751000
H	4.051600000	1.403517000	2.961812000
C	4.614283000	-0.023675000	1.429131000
H	5.658356000	-0.074460000	1.714060000
C	4.143293000	-0.781528000	0.368305000
H	4.815312000	-1.428664000	-0.178704000
C	2.793904000	-0.715527000	0.002029000
C	2.152506000	-1.471192000	-1.074250000
C	2.838918000	-2.375142000	-1.899103000
H	3.904004000	-2.537336000	-1.774193000
C	2.157890000	-3.073804000	-2.887332000
H	2.687560000	-3.772433000	-3.524749000
C	0.784206000	-2.869571000	-3.048412000
H	0.245554000	-3.416812000	-3.815820000
C	0.099329000	-1.970825000	-2.232605000
H	-0.966272000	-1.837016000	-2.383394000
C	0.757644000	-1.241642000	-1.229233000
C	-2.403855000	0.833274000	-1.722909000
H	-1.668088000	1.446542000	-2.225020000
C	-3.728267000	0.799140000	-2.124765000
H	-4.051582000	1.403533000	-2.961831000

C	-4.614281000	-0.023642000	-1.429141000
H	-5.658354000	-0.074422000	-1.714074000
C	-4.143301000	-0.781491000	-0.368308000
H	-4.815327000	-1.428618000	0.178704000
C	-2.793913000	-0.715498000	-0.002028000
C	-2.152524000	-1.471159000	1.074259000
C	-2.838947000	-2.375093000	1.899121000
H	-3.904035000	-2.537276000	1.774212000
C	-2.157927000	-3.073753000	2.887358000
H	-2.687606000	-3.772369000	3.524782000
C	-0.784241000	-2.869534000	3.048437000
H	-0.245596000	-3.416774000	3.815851000
C	-0.099354000	-1.970803000	2.232621000
H	0.966249000	-1.837005000	2.383409000
C	-0.757660000	-1.241624000	1.229242000
H	-2.204486000	3.657826000	2.336550000
H	-0.658654000	3.633368000	3.175203000
H	-1.016641000	4.943777000	2.024568000
